# The Liver in Children With Metabolic Syndrome

**DOI:** 10.3389/fendo.2019.00514

**Published:** 2019-08-02

**Authors:** Ebe D'Adamo, Valeria Castorani, Valerio Nobili

**Affiliations:** ^1^Department of Neonatology, University of Chieti, Chieti, Italy; ^2^Department of Pediatrics, University of Chieti, Chieti, Italy; ^3^Department of Pediatrics, University “La Sapienza”, Rome, Italy; ^4^Hepatology, Gastroenterology and Nutrition Unit, IRCCS “Bambino Gesù” Children's Hospital, Rome, Italy

**Keywords:** non-alcoholic fatty liver disease, metabolic syndrome, insulin resistance, childhood obesity, children

## Abstract

Non-alcoholic fatty liver disease (NAFLD) is recognized as an emerging health risk in obese children and adolescents. NAFLD represents a wide spectrum of liver conditions, ranging from asymptomatic steatosis to steatohepatitis. The growing prevalence of fatty liver disease in children is associated with an increased risk of metabolic and cardiovascular complications. NAFLD is considered the hepatic manifestation of Metabolic Syndrome (MetS) and several lines of evidence have reported that children with NAFLD present one or more features of MetS. The pathogenetic mechanisms explaining the interrelationships between fatty liver disease and MetS are not clearly understood. Altough central obesity and insulin resistance seem to represent the core of the pathophysiology in both diseases, genetic susceptibility and enviromental triggers are emerging as crucial components promoting the development of NAFLD and MetS in children. In the present review we have identified and summarizied studies discussing current pathogenetic data of the association between NAFLD and MetS in children.

## Introduction

In parallel with increasing prevalence of childhood obesity, non-alcoholic fatty liver disease (NAFLD) is becoming one of the most common health problems in obese children and adolescents (1). Hepatic steatosis is a clinical condition characterized by fat infiltration in more than 5% of hepatocytes in liver biopsy which is not related to excessive alcohol intake, autoimmune disease, viral infections or the use of steatogenic drugs (2). NAFLD encompasses a spectrum of histological hepatic alterations, ranging from hepatic steatosis to non-alcoholic steatohepatitis (NASH), fibrosis, cirrhosis, end-stage liver disease, and hepatocellular carcinoma (3).

Available data are strongly suggestive that morbidity of NAFLD extends beyond the liver and is associated with the major components of metabolic syndrome (MetS) already in pediatric age (4, 5).

First described in 1988 (6), MetS has been defined as a constellation of metabolic abnormalities and cardiovascular risk factors. Although MetS has received several definitions in pediatric population, there is agreement with respect to consider central obesity, impaired glucose tolerance, dyslipidemia, and hypertension as essential features (7) and, more importantly, NAFLD as the hepatic manifestation of this dysmetabolic state (8).

The molecular mechanisms linking NAFLD and MetS are controversial and the association of NAFLD with the main diagnostic criteria of MetS opens the debate as to whether hepatic steatosis is a consequence or a cause of this disease (9).

In the present review, we provide an overview of the most recent epidemiological and pathophysiological data of NAFLD and of the most common pathogenetic mechanisms linking NAFLD and MetS in childhood.

## Research Strategy

This systematic review is based on articles published over the past 10 years. A PubMed/MEDLINE search of literature was performed using the following key words: “Non-alcoholic fatty liver disease,” “Metabolic syndrome,” “Insulin resistance,” “Central Obesity,” “Ectopic fat deposition,” “Dyslipidemia,” “Genetic predisposition to metabolic diseases,” “Pathogenesis of NAFLD and MetS,” and “children.” During the research process bibliographic updates were perfomed.

## Epidemiology

In the last 20 years the prevalence of NAFLD has more than doubled in childhood. This escalation is partly due to the strong contribution of the growing rate of obesity in children, making NAFLD the most common cause of hepatic disease.

In the Unites States, 7 million children and adolescents are affected by hepatic steatosis (10) with a prevalence of 3–10% in the American population (11). Different prevalence data have been reported in Europe, ranging from 2.5% in UK (12) to 12.5% in Italy (13).

It is known that the presence of NAFLD increases with increasing body mass index (BMI) category. In an autoptic study conducted in California, the prevalence of histologically defined NAFLD was 38% in obese children (10). Accordingly, in a large community-based autoptic study, the prevalence of fatty liver disease was 26% in pediatric obese subjects (14). Furthermore, in a recent systematic review, Anderson et al. (15) have reported a prevalence rate of pediatric NAFLD ranging from 8% in non-obese children to 34% in obese children.

Different epidemiological studies (15, 16) reported that NAFLD appears twice as often in boys than in girls. In particular, Sartorio et al. (17) observed an higher prevalence of NAFLD in males than in females. Similar results have also been obtained in Asian and American obese children where hepatic steatosis was documented more frequently in boys than in girls (18–22). In the study by Gupta et al. (23), 22% of males were affected by NAFLD compared to 9.8% of girls. In accordance with the latest studies, Yu et al. (14) have recently observed an estimated NAFLD prevalence of 29.4% in males and 22.6% in females. Of note, in accordance with previous study (24), El-Karaksy et al. (25) and Di Bonito et al. (26) observed higher prevalence of abnormal serum alanine aminotransferase (ALT) levels in girls (55%) than in boys (36%). Also in chinese obese children, it was identified NAFLD more often in female than in male (27). The significant variance documented across the reported studies is partly explicated by the technique used to diagnosed NAFLD and probably by the influence of the pubertal development in which sex hormones could influence the onset of NAFLD (15, 28).

It is important to highlight that the prevalence of fatty liver disease varies widely also depending on region and ethnic descent. NAFLD is more frequent in Asian population than in Europe, Middle East/North Africa, and North America (15). South-American data have reported a prevalence of NAFLD of 2.3% in obese Brazilian children (29) and of 16% in Chilean pediatric population with obesity (30). Furthermore, NAFLD has been observed in 62.5% of obese Indian adolescents (31) and in 45% of obese Chinese children and adolescents (32).

Based on ethnicity, several studies observed that African Americans are less and Hispanics are more predisposed to develop fatty liver disease than Caucasians (33, 34). In accordance with evidence in adults (35, 36), in the prospective cohort study performed by Tricò et al. (37), 59.6% of Hispanic, 42.9% of white, and 15.7% of black obese adolescents were affected by NAFLD.

Of note, as suggested by Lomonaco et al. (38) in many epidemiological reports the ethnic groups were not matched for the major unfavorable metabolic factors, usually observed in Hispanics patients. By recruiting 152 Hispanics and Caucasian patients with biopsy-proven NASH matching for age, sex, and total body fat, the authors (38) observed a higher but not significant trend of liver fat content in Hispanic than Caucasian patients, without differences in the severity of NASH. Interestingly, when Hispanic and Caucasian subjects are matched for obesity, NASH ethnic differences between the two groups disappear, underlining how it's important to take into account the metabolic profile of patients.

Another potential factors explaining the wide range of steatosis prevalence is the test utilized for the diagnosis, based on liver histology, imaging studies, and evaluation of liver enzymes.

In the autopsy study conducted by Schwimmer et al. (10) the prevalence of pediatric NAFLD ranged from 0.7% in 2- to 4-year-old to 17.3% in 15-to 19-year-old subjects, with an increase to 38% in obese children.

By using imaging methods, Franzese et al. (39) have conducted the first study on the incidence of fatty liver disease as assessed by ultrasound. Liver steatosis was detected in 52% of obese Italian children. In accordance with this previous study, in recent evidence NAFLD was detected in 41% of obese pediatric population with liver ultrasonography (40) and 41.6% (37) with liver magnetic resonance imaging (MRI).

Different results have been reported measuring ALT levels, with a NAFLD prevalence ranging from 8 to 42% (41).

Although liver biopsy remains the gold standard method to diagnose NAFLD, it's invasive in children and not feasible in population study (2).

The currently recommended first-line screening test for fatty liver disease in children is ALT (42). ALT is an inexpensive, minimally invasive and universally available blood test with an acceptable sensitivity (41). Several studies in children have evaluated the upper limits of normal ALT (43–45). In the US, the ALT cutoffs are 22 mg/dl for girls and 26 mg/dl for boys (41). In a Canadian study the upper normal limit of normal ALT is 30 mg/dl in children between 1 and 12 years of age and 24 mg/dl in those between 13 and 19 years (44). For the diagnosis of NAFLD in obese pediatric population with >10 years of age, the threshold for ALT is ≥50 for boys and ≥44 for girls, while NASH has been observed in children with ALT ≥80 (45).

Recently, Bussler et al. (46) have provided new age- and sex-related percentiles for ALT in pediatric population. During early adolescence, the median of ALT serum concentration varies between 14.0 and 20.3 U/L in girls and between 17.1 and 21.1 U/L in boys. The 97th percentile spans from 24.2 to 31.7 U/L in girls and from 29.9 to 38.0 U/L in boys and children with ALT values >97th percentile present high risk of NAFLD.

Interestingly, the authors documented a significant positive correlation between BMI-SD score (BMI-SDS) and ALT serum concentrations. A rise of the BMI-SDS of around +1 results in an ALT serum level increase of around 2.21 U/L in boys and around 1.11 U/L in girls.

## Pathogenesis of NAFLD

Despite the advances in understanding the pathogenesis of NAFLD, the exact mechanisms underlying the development of fatty liver disease are still unclear.

The first and the most accepted hypothesis to describe the pathophysiology of NAFLD is the “two hits” theory (2) according to which liver damage is developed through two main components. In the “first hit” obesity, high-fat diet and insulin resistance (IR) seem to be responsible for the deposition of triglycerides (TG) in the hepatocytes, a pre-requisite for hepatocyte injury (2, 47). In the “second hit” oxidative stress, inflammatory cytokines, adipokines, mitochondrial dysfunction, and endoplasmic reticulum stress trigger the progression to NASH (2, 47).

During the last few years, this traditional model has been modified by the “multiple parallel hits” hypothesis (48), according to which NAFLD is defined as an epiphenomenon of several metabolic mechanisms involves genetic and enviromental factors as well as an inter-organ crosstalk between liver, adipose tissue, pancreas, and gut (47).

The pathogenesis of NAFLD seems to start in the intrauterine life, during which maternal BMI, MetS, gestational diabetes and low birth weight during pregnancy have been identified as prenatal risk factors (49, 50).

Furthermore, a genetic background characterized by genes involved in the main metabolic processes has been shown to predispose to NAFLD development (48, 51).

After birth, obesity and IR still represent the main factors involved in the first accumulation of fat in the liver.

It is well-established that higher BMI values are associated with the development of fatty liver disease in children and that most obese children are not adherent to lifestyle modifications and hypocaloric diets (52).

As shown by Mager et al. (53) children with hepatic steatosis spent more than 65% of their leisure time in sedentary. Nobili et al. (54) have reported that a 2-year lifestyle intervention, including monthly dietary counseling and physical activity, is correlated with weight loss, reduced dyslipidemia and ALT levels and improvement in liver histology in children with biopsy proven NAFLD. In accordance with the latest study (54), Pozzato et al. (55) have evaluated the effect of a 1-year lifestyle intervention, including regular physical activity (30–45 min·day^−1^, aerobic exercise) with a normocaloric balanced diet. The authors observed that the prevalence of liver steatosis decreased significantly from 34.6 to 7.7% as well as the obesity degree, the triglycerides, and total cholesterol levels (55). Of note, physically active lifestyle improves insulin sensitivity through decreasing fasting insulin and increasing lean mass (56).

Thus, the adoption of a physically active lifestyle and gradual weight loss should be considered in the prevention and treatment of the development of NAFLD in obese children.

As recently illustrated by several authors (47–49), not only the degree of obesity but also fat distribution and the lipodystrophic state in obese patients are associated with an increased efflux of free fatty acids (FFA) to the liver that contribute to the development of IR (47). The “insulin resistance state” is a general concept that should be refined to systemic IR, characterized by altered blood glucose concentrations, adipose tissue IR, characterized by the inabilty of insulin to suppress lipolysis, and hepatic IR, in which there is an impairment of hepatic glucose production (48). Furthermore, in a state of IR there is an over-expression of the sterol regulatory element binding protein-1c (SREBP-1c) with a consequent upregulation of *de novo* lipogenesis, an impairment of FFA β-oxidation and of *very low-density lipoprotein* (VLDL) secretion, with a further increase of hepatic lipid accumulation (2, 47).The FFA overload in the hepatocytes together with the release of adipokines and proinflammatory cytokines coming from the adipose tissue cause lipotoxicity, with a consequent mitochondrial dysfunction, increase of cytotoxic reactive oxygen species (ROS) production and endoplasmatic reticulum stress (2, 57).

The important role of oxidative stress in the progression of NAFLD has been clearly demonstrated by several studies (48, 58–60). Compared to normal healthy subjects, NAFLD patients present elevated levels of ROS and lipid peroxidation products and decreased concentrations of anti oxidants enzymes such as superoxide dismutase (SOD), reactive carbonyl species, catalase, and anti oxidants compounds such as glutathione (61). Pirgon et al. (62) performed a cross-sectional study in pediatric population and observed that obese adolescents with NAFLD reported increased oxidative stress indeces compared to adolescents without NAFLD. The pathogenetic mechanism linking NAFLD and oxidative stress is characterized by the storage of lipids in hepatocytes that induces β-oxidation of fatty acids, which promotes ROS production leading to the oxidative damage of mitochondrial membrane (63). This further worsens oxidative damage conducing to hepatocellular death and NASH progression (64). The oxidative stress caused by fatty acid overload in hepatocytes result from mitochondria, peroxisomes and microsomes. IR significantly increases peroxisomal oxidation because insulin is the pivotal inhibitor of cytochrome P450 4A (CYP4A), a key enzyme in this pathway (65). This inhibition amplifies cytotoxic ROS and lipid peroxidation. These products can diffuse into the extracellular space, influence Kupffer cells and hepatic stellate cells (HSCs) and active the nuclear factor-κB (NF-κB) pathway, which determine the subsequent synthesis of tumor necrosis factor-α (TNF-α) and several other proinflammatory and fibrogenic cytokines (66). Among this inter-organ crosstalk, human gut microbiota has been recently identified as a crucial player in the pathogenesis of NAFLD (67–69). Intestinal dysbiosis, bacterial overgrowth, and impairment of mucosa permeability lead to an increased bacterial flow into the liver thus exposing the hepatic tissue to pathogen associated molecular patterns (PAMP) (48, 67). By linking specific pattern recognition receptors (PRRs) on hepatic immune cells, PAMP activate a pro-inflammatory cascade with a consequent increase of hepatic inflammation (67). Lipopolysaccharide (LPS), highly immunogenic component of gram-negative cell walls, is the most studied PAMP (2). Raised LPS and LPS-binding protein levels have been observed in children with NASH (70, 71).

Moreover, bacterial fermentation of food substrate may contribute to the severity of NAFLD by increasing endogenous alcohol production and short chain fatty acids (SCFA) and decreasing choline metabolism (67, 72, 73). In accordance with previous study (74), Pierri et al. (75) observed that children with NAFLD showed more fermenting bacteria, particularly Escherichia species, and enhanced serum alcohol levels. Thus, although several potential pathogenetic mechanisms of NAFLD are emerging, it must be ackowledged that one factor is insufficient to drive liver disease progression, confirming its multifactorial nature.

## Common Pathogenetic Mechanisms Linking NAFLD and MetS

Despite the correlation between hepatic steatosis and MetS is often reported, this relationship is complex and not completely understood, due to the multifacted pathogensis of both diseases (7, 76).

Several studies in children support that NAFLD is associated with several components of MetS, such as IR, central obesity and dyslipidemia (7). Furthermore, emerging data underline a pivotal role of genetic predisposition (50, 77) and intrauterine events (50, 77) in the pathogenesis of the metabolic derangements in the pediatric age group.

**Figure 1:**
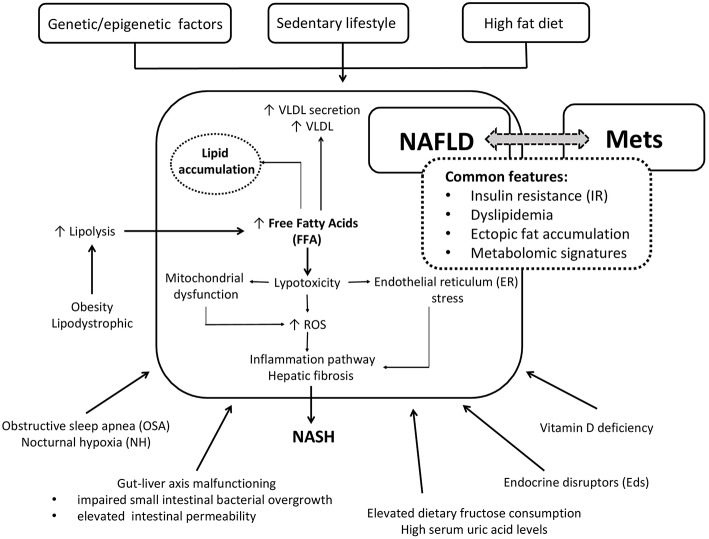
Simplified cartoon of the main pathophysiological mechanisms linking NAFLD and Mets in children.

The common patogenetic linking NAFLD and Mets are summarized in the Figure 1.

### Insulin Resistance (IR)

IR represents the critical connector linking NAFLD and MetS in obese children and adolescents (76).

As shown in the Table 1, this relationship has been observed in several studies (17–19, 21–27).

**Table 1:** Studies evaluating the association between insulin resistance, non-alcoholic fatty liver disease (NAFLD), and metabolic syndrome (MetS) in children.

**References**	**Study design**	**Sample size** **and** **Gender-NAFLD prevalence**	**Ethnic** **group**	**IR indexes**	**Findings**
Sartorio et al. (17)	Cross sectional study	268 obese children (58% M/42% F)	Caucasian	OGTT HOMA-IR	NAFLD is associated with impaired glucose profile and MetS criteria (systolic blood pressure, lipid profile, BMI).
Yoo et al. (18)	Cross-sectional study	909 obese children (78% M/22% F)	Asian	HOMA-IR Fasting insulin	Significant association between NAFLD and components of MetS (IR, dyslipidemia, ipertension)
Love-Osborne et al. (24)	Cross-sectional study	85 obese children (42% M/58% F)	Hispanic Black White American-Indian Asian	OGTT HOMA-IR Fasting insulin	Impaired glucose regulation and NAFLD in subjects meeting 3 or more criteria of MetS. Fasting insulin and TG were significantly higher in subjects with steatosis
Di Bonito et al. (26)	Cross-sectional study	564 obese and lean children (37% M/63% F)	Caucasian	HOMA-IR Fasting insulin Fasting glucose	A positive correlation between ALT levels, IR, and dyslipidemia
Shi et al. (27)	Cross-sectional study	308 obese children (46% M/76% F)	Asian	HOMA-IR WBISI	High prevalence of NAFLD in children with MetS. NAFLD and MetS shared the common mechanism of IR.
Fu et al. (19)	Cross-sectional study	861 obese children (70% M/30% F)	Asian	OGTT HOMA-IR Fasting insulin Fasting glucose HbA1c WBISI	Association between NAFLD, hypertension, dyslipidemia, impaired fasting glucose, and MetS. The state of insulin resistance deteriorated as the degree of fatty infiltration increased.
Boyraz et al. (22)	Cross-sectional study	451 obese children (65% M/35% F)	Caucasian	OGTT Fasting glucose Hyperinsulinemia	Association between NAFLD, MetS criteria, and IR.
Mager et al. (20)	Cross-sectional study	46 obese and lean children (94% M/6% F)	Canadian	HOMA-IR	Metabolic dysregulation (insulin resistance, obesity, elevated TG, and lower HDL cholesterol) in children with NAFLD.
El-Karaksy et al. (25)	Cross-sectional study	76 children overweight/obese (31% M/69% F)	Egyptians	QUICKI HOMA-IR	36.8% of children with NAFLD presented MetS. IR was higher in children with NAFLD than controls.
Lee et al. (21)	Cross-sectional study	12 obese children (58% M/42% F)	Black White	3-h hyperinsulinemic-euglycemic clamp	Association between NAFLD, IR, and adverse cardiometabolic profile.

*NAFLD, non-alcoholic fatty liver disease; MetS, metabolic syndrome; BMI, body mass index; ALT, alanine aminotransferase; IR, insulin resistance; OGTT, oral glucose tolerance test; HOMA-IR, homeostatic model assessment of insulin resistance; WBISI, whole body insulin sensitivity; QUICKI, quantitative insulin-sensitivity check index; HbA1c, hemoglobin A1c; TG, triglycerides; HDL, high-density lipoprotein; M, males; F, females*.

The strict connection between fatty liver disease and IR has been clearly documented by Caprio et al. (78). To evaluate the independent contribution of the liver in the alteration of insulin sensitivity, the authors have studied obese adolescents with similar overall degree of obesity and intramyocellular lipid content (IMCL) and different hepatic fat fraction. To note, hepatic steatosis was related to impaired insulin sensitivity and secretion (48), leading to the development of metabolic alterations. By recruiting 254 children and adolescents aged 6–17 years, Patton et al. (79) have demonstrated that the severity of IR was significantly correlated with histological features of NAFLD and the risk of MetS was greater among those with severe steatosis. In accordance with previous studies (40, 80), Prokopowicz et al. (81) observed an impaired metabolic profile, characterized by greater waist circumference, IR, glucose dysregulation, and dyslipidemia in 45% of overweight adolescents with hepatic steatosis than children without. Moreover, 40.8% of children with NAFLD presented MetS. These results have been further confirmed in the prospective study conducted by Tricò et al. (37) in a multiethnic cohort of obese adolescents. Particularly, authors underlined the association between high hepatic fat fraction, impaired insulin sensitivity and the development of metabolic alterations in children. The association between IR, NAFLD, and MetS has been also confirmed by using ALT levels as marker of hepatic steatosis. In a recent cross-sectional study of 77 obese non-diabetic children, Hampe et al. (82) have demonstrated that the frequency of high ALT levels increased paralleling to the increased number of MetS diagnostic criteria.

To note, the influence of IR on the metabolic parameters persists over time. By following for an average of 1.9 years 76 obese youths, Kim et al. (83) observed a persistence of IR state in children with increased hepatic fat fraction and an improvement of insulin sensitivity index and glucose metabolism in children without fatty liver.

Thus, independently of the diagnostic tool and the age of the patients, IR represents a key factor linking the development of MetS features in children with liver steatosis.

### Ectopic Fat Accumulation

During the last few years, several studies have shown that altered partioning of fat plays a pivotal role in the development of IR, NAFLD, and MetS in obese children children and adolescents (84).

As clearly demonstrated by Caprio et al. (85), increased visceral adipose tissue (VAT) and a thin superficial layer of abdominal subcutaneous fat (SAT) are associated with the propensity to store fat in the liver and muscle leading to IR and the MetS (86). By stratifyng a multiethnic cohort of obese adolescents into tertiles based on the proportion of abdominal fat, the authors have demonstrated that the increased proportion of VAT and the decreased percentage of SAT were associated with muscle and hepatic steatosis, IR and a higher risk of MetS in obese adolescents with this phenotype than those without (87).

The association between higher VAT proportion, ectopic liver fat accumulation, and metabolic alterations seem to persist over time. In a longitudinal evaluation of 151 obese adolescents, Umano et al. (88) have recently demonstrated that baseline VAT/(VAT + SAT) ratio represents a predictor of changes in intrahepatic fat content, degree of IR, and impaired glucose metabolisms at follow-up among girls. Moreover, the prevalence of MetS at follow-up was higher in obese girls with high proportion of VAT to abdominal SAT than those with a low ratio.

Based on this “lipodistrofy model,” the poor expandability of SAT might lead to an excess accumulation of lipids in the VAT, which represents a major source of proinflammatory mediators and FFA, the two major players in the development of fatty liver and IR (88).

To understand the mechanisms responsible for the inefficient storage of fat in the abdominal SAT, Kursawe et al. (89) have evaluated the fat cell size in a group of obese adolescents with similar degree of obesity and different VAT/SAT ratio. Obese subjects with high VAT/(VAT/SAT) ratio were characterized by a low fraction of large adipocytes showing an increased diameter and a greater proportion of the smaller adipose cells. In contrast, a more homogenous cellularity has been observed in the obese adolescents with a low VAT/SAT ratio. Thus, the hypertrophy of the largest cells and the reduced capacity of the smaller adipocytes to store fat might explain the high plasma levels of FFA and the associated fatty liver disease in obese adolescents.

Moreover, to evaluate the relationship between alterated fat partioning and the development of impaired glucose metabolism and IR, the authors have investigated the expression of lipogenic genes in abdominal subcutaneous adipose and liver tissue of 53 adolescents with similar degree of obesity across the spectrum of glucose tolerance (90). The expression of carbohydrate-responsive element binding protein (ChREBP) was decreased in the SAT of adolescents with impaired glucose tolerance and increased in the liver, explaining the association between upregulation of lipogenesis and NAFLD (90).

Thus, independent of the overall body fat mass, an impaired abdominal fat distribution represents a key factor in the pathogenesis of IR, fatty liver disease and MetS in the pediatric age.

### Dyslipidemia

There is a growing body of evidence suggesting an association between NAFLD and atherogenic lipid profile (91). Studies in adults (92, 93) have shown that increasing grades of NAFLD are significantly associated with increasing levels of serum total cholesterol, low-density lipoprotein cholesterol (LDL) and VLDL levels and with decreasing concentration of high-density lipoprotein cholesterol (HDL) (93).

Recently Zheng et al. (94) performed a cross-sectional study confirming that NAFLD is positively correlated with atherogenic lipid profile and patients enrolled with NAFLD shown carotid intima-media thickness (CIMT) and elevated brachial-ankle pulse wave velocity (ba-PWV) for stiffening.

Over the last decade, an increased incidence of alterations in lipid profile has also been documented in obese youth (95). In American population study, the prevalence of high total cholesterol among children and adolescents has been estimated to be 7.4%, with a higher rate among subjects aged 16–19 (8.9%) than in children aged 6–8 (6.0%) (96). In Italy, it was reported that 15% of children present a lipid profile classifiable as atherogenic dyslipidemia (97).

Dyslipidemia is a crucial diagnostic criterion of MetS but there is increasing evidence proving a close association between impaired lipid profile and NAFLD in children and adolescents.

In 2010, for the first time, Nobili et al. (98) have shown that in children with NAFLD the severity of liver injury was strongly associated with the presence of a more atherogenic lipid profile measured as TG/HDL-C, total cholesterol/HDL-C, and LDL/HDL-C ratios. TG/HDL-C ratio is a powerful marker of atherogenesis induced by IR and is associated with a more atherogenic lipid profile and early cardiovascular events (99, 100).

In 2015, Corey et al. (101) obtained similar results observing that dyslipidemia, characterized by elevated non-HDL-C levels and increased TG/HDL-C, is frequent in children and adolescents with NAFLD. Furthermore, the authors observed that both resolution and histologic improvement of NASH were associated with the improvement of non-HDL-C, LDL, and total cholesterol levels.

By evaluating a cohort of children and adolescents with NAFLD, Pacifico et al. (102) have demonstrated that subjects with a high triglycerides/HDL-C ratio have an increased risk of IR and an association between this ratio and NAFLD.

In accordance with previous studies (103), Balanescu et al. (104) have recently documented that NAFLD patients presented a very high TG/HDL-C ratio and a tendency to hyperuricemia and lower HDL levels compared with non-NAFLD patients.

Thus, TG/HDL-C ratio might represent a useful cardiovascular risk marker in pediatric patients with NAFLD.

Recently, Dowla et al. (105) by evaluating 309 children with NAFLD have observed elevated levels of TG, non-HDL-C and LDL-C and low serum concentrations of HDL-C in children wth NAFLD compared to healthy youths. Moreover, the authors documented a correlation between systolic blood pressure (SBP), insulin, glucose, hemoglobin A1c (HbA1c), liver enzyme [aspartate transaminase (AST)] and serum TG and non-HDL-C.

In accordance with other studies (106), Lee et al. (107) enrolled 548 obese pediatric subjects with NAFLD to investigate their anthropometric parameters and correlate risk factors presenting with hepatic steatosis. The authors observed that mean TG levels were in proportion to NAFLD severity.

Thus, lipid profile is an emerging risk factor in the development of cardiovascular diseases and in the pathophysiology of pediatric NAFLD and metabolic disorders.

### New Potential Pathogenetic Triggers of NAFLD

#### Vitamin D

In addition to the discussed pathogenetic mechanisms, Vitamin D levels have been recently identified as a risk factor for metabolic alterations in children. Poor Vitamin D status has been previously correlated with childhood obesity and insulin IR (108, 109). In a cross-sectional study Turer et al. (110) observed a significantly greater prevalence of Vitamin D deficiency in overweight, obese, and severely obese US children than controls. Furthermore, Olson et al. (111) observed a significant inverse relationship between 25(OH)D levels and Homeostatic Model Assessment of Insulin Resistance (HOMA-IR) in obese children.

A growing body of evidence has recently demonstrated an association between Vitamin D levels and pediatric NAFLD (112). Vitamin D deficiency has been correlated with a high risk of steatosis, necroinflammation, and fibrosis in adults (113, 114) as well as in children with biopsy-proven NAFLD (115). Nobili et al. (116) observed an inverse association between serum level of Vitamin D and histological liver damage in children with NAFLD, which also presented elevated BMI, dyslipidemia, impaired glucose metabolism, and hypertension. In accordance with the latest study (116), Black et al. (117) have documented a significant correlation between 25(OH)Vitamin D concentrations and hepatic fibrosis in a cohort of Australian children with hepatic steatosis. Moreover, children with NAFLD have shown more features of MetS, such as IR, impaired lipid profile, and elevated waist circumference than healthy controls.

The exact mechanisms by which Vitamin D influcences the development of NAFLD is still unclear. However, current data suggest that Vitamin D modulates fibrotic mechanisms in the liver by inhibiting the expression of transforming growth factor beta (TGF- β). Therefore, Vitamin D suppresses the deposition of collagen Iα1 and activates the alpha-smooth muscle actine (α-SMA) in HSCs influencing the development of hepatic steatosis (118). Studies in rats have also documented that Vitamin D deficiency upregulates hepatic inflammatory genes (119, 120) with a consequent NAFLD exacerbation.

Moreover, many studies (121, 122) highlighted the role of gene variants in the modulation of Vitamin D concentrations. Gibson et al. (123) performed the first study showing a relationship between genetic variations of Vitamin D receptor (rs2228570 polymorphism) and the severity of liver histology in UK children with NAFLD.

To note, several authors have also proposed a close link between Vitamin D deficiency and Mets in children and adolescents (124). In 2011 Ganji et al. (125) have documented that Vitamin D levels were inversely related to the prevalence of MetS phenotype, waist circumferance and systolic blood pressure in children. Moreover, the authors observed a direct association between 25(OH)D and HDL cholesterol levels. Accordingly, Lee et al. (126), reported a cross-sectional association between Vitamin D levels and MetS among 1,660 Korean children. In a Caucasian pediatric cohort, Pacifico et al. (127) concluded that central obesity, hypertension, hypertriglyceridemia, low HDL cholesterol levels, and MetS were correlated with low 25(OH)D levels.

Based on these findings, several studies (128, 129) have evaluated the possible therapeutic role of diet interventions in children with liver steatosis. In a randomized control- trial (RCT), Della Corte et al. (130) have observed that administration of docosahexaenoic acid (DHA) and Vitamin D reduced steatosis, ballooning and lobular inflammation and improve IR in a cohort of obese Caucasian children.

Thus, Vitamin D levels should be investigated in obese children with metabolic complications.

#### Obstructive Sleep Apnea (OSA)

Recently, increasing studies suggested that nocturnal hypoxia (NH) associated with obstructive sleep apnea (OSA) may promote the progression of pediatric NAFLD (131). In 2016, Sundaram et al. (132) observed a strict correlation between OSA/hypoxia in obese adolescents and advanced NAFLD histology and an increased systemic and hepatic oxidative stress with a reduction of circulating antioxidant agents. The study highlighted how low oxygen levels could be an important trigger of oxidative stress that promotes progression to NASH.

Interestingly, Paschetta et al. (133) reported that, during hypoxia, many transcription factors, including hypoxia-inducible factors (HIFs), modulate target genes expression that are involved in hepatic disease. In particular, the chronic intermittent cycles of hypoxia and reoxygenation (CHI) increase HIFs levels, which regulates lipogenesis and triglycerides storage with increased fatty acid synthesis in hepatocytes (134). To note, Nobili et al. (135) revealed that the presence and severity of OSA are associated with significant fibrosis and with NAFLD activity score in children, independently of body mass index, abdominal adiposity, metabolic syndrome, and IR. This relationship held also in non-obese subjects with NAFLD. Thus, in pediatric NAFLD, OSA could modulate biochemical, immunohistochemical, and histological features of NAFLD, NASH, and fibrosis.

#### Uric Acid and Fructose

Actually, there is a growing body of evidence suggesting that high serum uric acid (SUA) level is an independent risk factor of NAFLD (136, 137). The pathological mechanism linking NAFLD and hyperuricemia is not completely understood (138–140). Experimental data suggested that SUA is able to determine systemic inflammation, endothelial dysfunction and IR (141, 142) and to induce ROS (143) and activation of the NOD-like receptor family and pyrin domain containing 3 (NLRP3) inflammasome (144).

In line with recent cross-sectional studies in adults (145, 146), Vos et al. (147) examined the relationship between SUA levels and NAFLD in the pediatric population, reporting that uric acid was significantly increased in those children with histological diagnosis of NASH compared with milder forms of NAFLD. In accordance with the latest study, Nobili et al. (148) have recently observed that hyperuricemia, coupled with elevated dietary fructose consumption, could be responsible for the different pattern of lobular disease observed in pediatric NAFLD, determining greater damage in the periportal zone rather than in perivenous zone (148). Similarly, in a pilot study by Sullivan et al. (149) children with NAFLD after fructose ingestion reported an exacerbated metabolic profile characterized by elevated serum glucose, insulin, and uric acid associated with an higher urinary levels of uric acid and a lower fructose excretion than lean subjects. The authors hypothetized that children with NAFLD may be both absorbing and metabolizing fructose more than lean subjects, which could contribute to the pathophysiology of hepatic steatosis (149). On the contrary, the restriction of fructose for 9 days determined a decrease of liver fat and *de novo* lipogenesis (150).

Of note, these studies suggest a correlation between high fructose consumption, SUA, and NAFLD (151). In 2017, Mosca et al. (152) reported that dietary fructose consumption was positively and independently associated with hyperuricemia and that SUA concentrations and fructose intake are independently and positively associated with NASH in a cohort of children and adolescents with a histological diagnosis of NAFLD.

Thus, many data have identified the role of hyperuricemia in NAFLD, suggesting that SUA may be a new potential pathogenetic trigger for NAFLD patients.

#### Endocrine Disruptors

Endocrine disruptors (EDs) are “exogenous substances or mixtures that modify the function of the endocrine system, determining adverse effects on the health of an organism, or its progeny, or (sub)population” (153, 154).

During the last decade, several studies (155, 156) have demonstrated the relationship between EDs and the progression of metabolic and cardiovascular diseases, including obesity, IR, type 2 diabetes, hepatic injury, and dyslipidemia (157). In these alterations, EDs interact with the peroxisome proliferator-activated receptors (PPARs), with sex steroid and with corticosteroid receptors, influencing the cortisol sex steroid metabolism or induce epigenetic alterations promoting adipogenesis and lipid accumulation (158).

Over the past four decades, several EDs have been mass produced due to their widespread use. Among them, bisphenol A (BPA) is considered an EDs that largely promote cardiovascular and metabolic dysfunction (159). It is a weakly estrogenic chemical that is largely employed in manufacturing polycarbonate plastics, in food packaging and in toys (160). Animal studies reported that the perinatal exposure to BPA predisposed to increased body weight, elevated serum insulin, and impaired glucose tolerance in adult's offspring. Moreover, high fat-fed offspring exposed to BPA presented obesity, hyperglycemia, hyperleptinemia, hyperinsulinemia, and dyslipidemia (161). Menale et al. (162) observed a direct relationship between BPA and insulin resistance in a cohort of obese children suggesting that BPA exerts a suppression of adiponectin gene expression, leading to a reduction of adiponectin release and an increased of resistin expression.

Recently, increasing data have provided evidence that BPA exposure enhances susceptibility to NAFLD (157). As reported by Wei et al. (163), in an animal study an high-fat diet and BPA-exposition, are associated with the severity of NAFLD, increased liver triglycerides, liver free fatty acids, and serum ALT levels.

Khalil et al. (164) performed a cross sectional study in obese and overweight children and demonstrated an association between urinary BPA (creatinine), elevated liver enzyme and diastolic blood pressure even after adjusting for age and ethnicity. Interestingly, also in male children, serum fasting insulin (FI) and HOMA-IR showed a non-linear association with increasing urinary BPA concentration (164). Recently, Verstraete et al. (165) performed the first cross-sectional study in children that evaluated the association between BPA and suspected NAFLD, defined as elevated ALT levels, overweight or obesity and evidence of IR. The authors observed that the risk of NAFLD was increased in the higher quartile of BPA urinary levels (165).

Thus, bisphenol A exposure represents a potential risk factor for the initiation and progression of NAFLD.

### Genetic Susceptibility

Genetic pre-disposition and epigenetic mechanisms are emerging as one of the most important risk factors in the development and progression of NAFLD and MetS.

Genetic predisposition is suggested by documented familial clustering of NAFLD, as demonstrated by the ethnic differences in the prevalence of these disorders. Furthermore, not all the obese patients develop NAFLD suggesting the complex interplay between environmental factors and genetic predisposition (51).

Recently, several genetic variants in the DNA code (single DNA base-pair or single nucleotide polymorphism-SNPs) have been identified as potential markers for either susceptibility and progression of MetS and NAFLD (see Table 2) (166–172).

**Table 2:** Genes implicated in the development of NAFLD and MetS in children.

**Gene**	**Protein**	**Polymorphism**	**Function**
*PNPLA3* (166)	Adiponutrin/patatin-like phospholise domain-containig protein 3	*rs738409*	Enzyme (lipase) that mediates triacylglycerol hydrolysis in adipocytes. The protein may be involved in the balance of energy usage/storage in adipocytes.
*GCKR* (167)	Glucokinase Regulatory Protein	*rs1260326 rs780094*	Protein produced in hepatocytes. GCKR binds and moves glucokinase (GK), thereby controlling both activity and intracellular location of this key glucose metabolism enzyme.
*UCP3* (168)	Uncoupling protein 3	*rs11235972*	The uncoupling protein is involved in the transferring of anions from inner mitochondrial membrane to outer mitochondrial membrane, its protein is programmed in a way to protect mitochondria from induced oxidative stress.
*TM6SF2* (169)	Transmembrane 6 superfamily member protein 2	*rs58542926*	The physio-pathological function is not yet completely known. May function as sterol isomerase and as regulator of liver fat metabolism influencing triglyceride secretion and hepatic lipid droplet content.
*MBOAT7* (170)	Membrane bound O -acyltransferase domain containing protein 7	*rs626283*	Enzyme that have acyltransferase activity. This protein is involved in the re-acylation of phospholipids and it is implicated in arachidonic acid recycling in human neutrophils.
*PPARGC1A* (171)	Peroxisome proliferator–activated receptor-γ coactivator (PGC)-1α protein	*rs8192678*	Master transcriptional coactivator that module multiple key genes that play a role in energy homeostasis, mostly through the control of mitochondrial function and biogenesis.
*MTTP* (172)	Microsomal triglyceride transfer protein	*rs2306986*	Carrier-protein that is involved in lipid transfer function. It plays a pivotal role critical in the assembly and secretion of very-low-density lipoprotein (VLDL) removing lipid from liver.

*PNPLA3, adiponutrin/patatin-like phospholipase domain-containing 3; GCKR, glucokinase regulatory protein gene; UCP3, uncoupling protein 3; TM6SF2, transmembrane 6 superfamily member 2; MBOAT7, membrane bound O -acyltransferase domain containing protein 7; PPARGC1A, peroxisome proliferator-activated receptor- γ coativator-1α; MTTP, microsomal triglyceride transfer protein*.

As shown by Santoro et al. (166) in a cohort of obese children and adolescents the rs738409 variant of *adiponutrin/patatin-like phospholipase domain-containing 3 (PNPLA3)* confers susceptibility to hepatic steatosis by determining elevated serum ALT levels. In accordance with the latest study, Mangge et al. (173) have observed that the PNPLA3 rs738409 polymorphism is correlated with increased ALT levels in pediatric age group and it's more frequent in obese children with MetS. PNPLA3 encodes for a hepatic enzyme, the adiponutrin, that is also present in adipose tissue showing both a lipogenic and lipolytic activity *in vitro* (174). This missense variant causes a cytosine to guanosine substitution determining an isoleucine to methionine substitution at the amino acid position 148 (I148M). Although the mechanisms underling the association with this variant and liver stetaosis is still debated, studies *in vitro* have shown that PNPLA3 rs738409 variant limits TG hydrolysis by inhibiting catalytic activity of the enzyme and increases the lipogenesis activity of the PNPLA3 (175). Furthermore, it has been shown that FFA potentiate the effect of PNPLA3 rs738409 variant on intra-hepatic triglycerides accumulation (176). Thus, PNPLA3 genotyping might become a test to characterize patients with NAFLD, however more studies are needed to clarify how this variant affects the development of fatty liver disease (177).

Another gene correlated with susceptibility and progression of NAFLD is the *glucokinase regulatory protein (GCKR) gene* which encodes for the glucokinase regulatory protein (GCKRP). The GCKRP regulates the activity of glucokinase (GCK) by competing with the glucose which is a substrate of GCK (167). The rs1260326 variant has been studied as potential marker of hepatic steatosis and MetS in children and adolescents. This variant consists of proline-to leucine substitution in position 446 (P446L) and encodes for a protein characterized by a reduced capability to response to fructose 6 phosphate, leading to a constant increased of GCK activity (4). In accordance with studies in adults (178, 179), Santoro et al. (180) have demonstrated that the GCK rs1260326 variant is associated with NAFLD, elevated triglycerides and large VLDL levels in a cohort of obese children. In accordance with the latest study, Lin et al. (181) have been documented a significant correlation between GCKR rs780094 genotype and some components of MetS, such as elevated serum level of cholesterol and triglycerides and high serum levels of ALT. To note, Hudert et al. (182) have shown that GCKR modules the amount of hepatic fibrosis in pediatric NAFLD.

Similarly, *uncoupling protein 3 (UCP3) gene* could influence the occurrence of NAFLD in pediatric population. This gene is located on chromosome 11q13 and is involved in the regulation of energy and lipid metabolism (168). In 2013, Xu et al. (183) have found that children with NAFLD were characterized by a higher prevalence of rs11235972 GG genotype compared with the health control group. The authors observed that the increase risk of NAFLD is significantly associated with BMI, waist-to-hip ratio (WHR), blood pressure (BP), fasting blood glucose (FBG), IR, and dyslipidemia.

The *Glu167Lys (E167K) transmembrane 6 superfamily member 2 (TM6SF2)* variant is a further genetic polymorphism associated with liver fibrosis, ALT levels and reduced liver-derived TG rich lipoproteins plasma levels (169). Grandone et al. (184) by evaluating an Italian cohort of obese children and adolescents have shown an association between TM6SF2 rs58542926 variant, liver enzymes and hepatic steatosis. Of note, it seems to reduce cardiovascular risk.

Another gene recently associated with liver function in the pediatric population is the *MBOAT7 gene*, that encodes for a lysophosphatidylinositol acyltransferase-1, known as LPIAT1 or MBOAT7 which plays a relevant role in arachidonic acid recycling in human neutrophils (170). In accordance with a previous study in adults (185–187), Umano et al. (188) have recently documented a significant correlation between a gene variant rs641738 in the MBOAT7 gene and hepatic steatosis in a multiethnic cohort of obese children and adolescents. Interestingly, they observed an association between the rs626283 SNP in the MBOAT7 gene and impaired insulin sensitivity only in Caucasian obese youths.

### Epigenetic Mechanisms

During the last decades several authors have postulated a strong association between epigenetic alterations in intrauterine life and the development of metabolic and cardiovascular complications later in life (50). These multifactorial mechanisms modulate gene expression of offspring (189) and consist in DNA methylation, remodeling of chromatin, histone modifications, and/or regulatory feedback by microRNAs. Studies in humans have demonstrated that these mechanisms contribute to the programming of NAFLD (50). Brandt et al. (190) have recently observed higher levels of miRNA-122 in obese children compared to healthy controls and a positive correlation between circulating miRNA122 levels and ALT, aspartate transaminase (AST), and gamma glutamil transpeptidasi (GGT) values in prepubertal children (190). On the contrary, in obesity mouse model inhibition of miR-122 activity results in decreased plasma cholesterol concentration with reduced expression of several lipogenic genes that significantly improve NAFLD (191). Probably, miR-122 might be useful as a new biochemical marker for assessing early hepatic steatosis (192).

Emerging evidence also supports a role of DNA methylation in the development of cardiometabolic disorders. Recently, Gerhard et al. (193) have observed significant differences in DNA methylation between adults with biopsy-proven of NAFLD and controls. Moreover, a different methylation pattern was documented in humans with advanced vs. mild grade of NAFLD (194). It's clear as differences in methylation profiles could modulate hepatic genes expression, thus influencing NAFLD pathogenesis. AQP1 (aquaporin 1) gene, encoding a water channel, and FGFR2 (fibroblast growth factor receptor 2) result overexpressed and hypomethylated in fibrosis condition (195, 196).

Alterations in DNA methylation and histone modifications have also been proposed to be important in the programming of NAFLD in offspring exposed to maternal obesity which causes altered expression of genes involved in gluconeogenesis and lipid metabolism (197). McCurdy et al. (198) have found that offspring from mothers chronically consuming a high-fat diet (HFD) had an increased liver triglycerides and an elevated expression of genes for mitochondrial oxidative stress or for fatty acid oxidation, with a premature activation of the gluconeogenic pathway that modulate the development of NAFLD. Recent studies in neonates have demonstrated a strict association between maternal BMI and intra-hepatocellular lipid levels assessed by magnetic resonance imaging (199, 200). Moreover, in the cross-sectional study conduceted by Ayonrinde et al. (201) in a cohort of adolescents aged 17 years, maternal obesity and adolescent obesity increased the risk of a hepatic steatosis independent of a Western dietary pattern, while breatfeeding reduced the risk of NAFLD. Of note, in 2019 Nguyen et al. (202) have shown that reduced sirtuin (SIRT)1 expression in offspring from mothers consuming HFD-dietinduces a pre-diabetic and NAFLD phenotype. SIRT1 is a potent regulator of energy metabolism and stress responses, express in the hypothalamus, white adipose tissues (WAT), and liver. A systemic overexpression of SIRT1, in the offspring from mothers consuming HFD-diet, determined a reduced body weight and adiposity in epididymal and retroperitoneal adipose tissues of offspring with normalized lipid metabolic markers and fibrogenesis in the liver (202).

Whether all these gene alterations are causative or simply a consequence of the induced disease state remains to be determined by other studies (203). However, these results indicate that maternal metabolic dysfunction influences NAFLD phenotype in offspring (204), underling the importance to encourage the maintenance a normal body mass index and an healthy diet before and during pregnancy.

### Metabolomic

Recent studies hypothesized that differences in serum and urinary metabolite profiles between healthy and obese children could be correlated with clinical phenotype and metabolic risk in pediatric population. Troisi et al. (205) performed a urinary metabolome analysis in 40 italian children and adolescents to identify specific metabolic pathways that characterize pediatric obese subjects with or without NAFLD. The authors observed lower urinary levels of xylitol and phenylacetic acid (PAA) in obese children (with and without NAFLD) than in normal weight controls (205). Xylitol, a five-carbon sugar alcohol, is present in many types of fruits and vegetables and it is not endogenously produced by humans. PAA is an organic compound synthesized from the amino acid phenylalanine (PA) via phenylpyruvate. Plant secondary metabolites such as phenolic acids are generally correlated with positive effects for human health (206). Thus, urinary levels of these metabolites reflect the diet preferences and they have been considered beneficial in preventing the development of obesity and metabolic alterations in rats with diet-induced obesity (207).

Interestingly, also high urinary concentration of methylhistidine, which derives from meat intake, have been obeserved in patients with NAFLD (205). This substance has been correlated with BMI in obese subjects, reflecting its association with muscle mass and dietary meat intake (208). 1-methylhistidinuria could originate also from increased oxidation in skeletal muscle, probably eased by a reduction in the body's antioxidants pool, e.g., by alpha-tocopherol deficiency, a condition largely documented in pediatric obesity-related NAFLD (209).

Other specific metabolic pathways have been associated with gut-liver axis malfunctioning which was frequently detected in obese children with NALFD. Indeed, urinary p-cresylsulphate (PCS) level, an intestinal microbial metabolite, resulted increased in obese children without NAFLD and correlated negatively with the presence of small intestinal bacterial overgrowth (SIBO) (205). Branched chain amino acids (BCAAs) and/or their metabolites were associated with excess of visceral fat, elevated intestinal permeability (IP) and SIBO (205).

Recently, metabolic salivary markers of hepato-comorbidities have been identified in pediatric obese subjects (210). Troisi et al. (210) performed a preliminary study that demonstrated high serum and salivary glucose and insulin levels in obese children with or without MetS.

In this study, it was also reported that salivary insulin concentration tended to be higher in obese patients with steatosis than in those without and that increased in parallel with the number of MetS elements (211).

Further studies have recently confirmed as different salivary metabolic profiles characterized pediatric obesity, liver disease, and MetS (212, 213). Troisi et al. (213) observed high levels of two saturated fatty acids, palmitic, and myristic acid, in children with steatosis. Wasilewska et al. (214) demonstrated a positive correlation between total ceramide concentration and IR markers in obese children with NAFLD. Moreover, Qi et al. (215) observed high levels of salivary pyroglutamic acid in patient with NAFLD, suggesting that it could be a potential diagnostic biomarker of enhanced oxidative stress in liver cells.

Thus, distinct metabolomic signatures have been identified to detect fatty liver and MetS in childhood. Metabolomic could represent a useful tool for identifying children at risk of obesity hepato/metabolic complications in epidemiologic evaluations.

## Conclusive Remarks and Future Perspectives

The present review confirms that the prevalence of pediatric NAFLD is increasing worldwide and there is emerging evidence supporting the relationship between fatty liver disease and MetS in children and adolescents.

The strict link between NAFLD and MetS in pediatric age group is becoming increasingly clear because these diseases are both associated with a high risk to develop cardiovascular and diabetic complications early in life.

Although central obesity and IR are still considered as the key factors linking MetS and NAFLD, a multifactorial pathogenesis characterized by the interaction between genetic background and environmental factors is increasingly recognized as a key player in the development of both metabolic disorders.

Actually, the mainstay of NAFLD therapy is characterized by lifestyle interventions on obesogenic environment and sedentary life, which the objective to reduce obesity-related hepatic and metabolic abnormalities. Unfortunately, this target is often difficult to be obtained and the results are not always satisfied. Based on the new identified potential pathogenetic triggers, novel pharmacologic therapies have been proposed such as probiotics that now seem the most remarkable and reasonable approach for their safety and acceptability.

In conclusion, children and adolescents with NAFLD should be screened for MetS as well as those with MetS for fatty liver disease.

Future perspectives must focus on understanding the natural history and etiology of NAFLD in order to identify early prevention stategies and efficient therapeutic approaches to improve the quality of life of these children.

## Author Contributions

All the authors contributed to the research of the articles and to the writing of the manuscript. VN revised it critically.

### Conflict of Interest Statement

The authors declare that the research was conducted in the absence of any commercial or financial relationships that could be construed as a potential conflict of interest. The handling Editor declared a shared affiliation, though no other collaboration, with several of the authors ED'A and VC.

## Abbreviations

NAFLD: non-alcoholic fatty liver disease
NASH: non-alcoholic steatohepatitis
MetS: metabolic syndrome
BMI: body mass index
ALT: alanine aminotransferase
MRI: magnetic resonance imaging
BMI-SDS: body mass index SD score
IR: insulin resistance
TG: triglycerides
FFA: free fatty acids
SREBP-1c: sterol regulatory element binding protein-1c
VLDL: very low-density lipoprotein
ROS: reactive oxygen species
SOD: superoxide dismutase
CYP4A: cytochrome P450
HSCs: hepatic stellate cells
NF-κB: nuclear factor- κB
TNF-α: tumor necrosis factor-α
PAMP: pathogen associated molecular patterns
PRRs: pattern recognition receptors
LPS: lipopolysaccharide
SCFA: short chain fatty acids
IMCL: intramyocellular lipid content
VAT: visceral adipose tissue
SAT: superficial layer of abdominal subcutaneous
ChREBP: carbohydrate-responsive element binding protein
HDL-C: high-density lipoprotein cholesterol: LDL, low-density lipoprotein cholesterol
CIMT: carotid intima-media thickness
ba-PWV: brachial-ankle pulse wave velocity
SBP: systolic blood pressure
HbA1c: Hemoglobin A1c
AST: aspartate transaminase
HOMA-IR: homeostatic model assessment of insulin resistance
TGF-β: transforming growth factor beta
α-SMA: alpha-smooth muscle
RCT: randomized control- trial
DHA: docosahexaenoic acid
OSA: obstructive sleep apnea
NH: nocturnal hypoxia
HIFs: hypoxia-inducible factors
CHI: hypoxia and reoxygeneration
SUA: seum uric acid
NOD-like receptor family: pyrin domain containing 3 (NLRP3)
EDs: endocrine disruptors
PPARs: peroxisome proliferator-activated receptors
BPA: bisphenol A
SNPs: single nucleotide polymorphism
PNPLA3: adiponutrin/patatin-like phospholipase domain-containing 3
GCKR: glucokinase regulatory protein gene
GCKRP: glucokinase regulatory protein
GCK: glucokinase
P446L: proline-to leucine substitution in position 446
UCP3: uncoupling protein 3
WHR: waist-to-hip ratio
BP: blood pressure
FBG: fasting blood glucose
TM6SF2: transmembrane 6 superfamily member 2
MBOAT7: Membrane bound O -acyltransferase domain containing protein 7
GGT: gamma glutamil transpeptidasi
AQP1: aquaporin 1
FGFR2: fibroblast growth factor receptor 2
HFD: high-fat diet
SIRT: sirtuin
WAT: white adipose tissues
PAA: phenylacetic acid
PA: amino acid phenylalanine
PCS: p-cresylsulphate
SIBO: small intestinal bacterial overgrowth
BCAAs: branched chain amino acids
IP: intestinal permeability.

## References

[1] Nobili V Svegliati-Baroni G Alisi A Miele L Valenti L Vajro P. A 360-degree overview of paediatric NAFLD: recent insights. J Hepatol. (2013) 58:1218–29. 10.1016/j.jhep.2012.12.003 23238106

[2] Mann JP Valenti L Scorletti E Byrne CD Nobili V. Nonalcoholic fatty liver disease in children. Semin Liver Dis. (2018) 38:1–13. 10.1055/s-0038-1627456 29471561

[3] Mann JP De Vito R Mosca A Alisi A Armstrong MJ Raponi M. Portal inflammation is independently associated with fibrosis and metabolic syndrome in pediatric nonalcoholic fatty liver disease. Hepatology. (2016) 63:745–53. 10.1002/hep.28374 26638195

[4] Mikolasevic I Milic S Turk Wensveen T Grgic I Jakopcic I Stimac D. Nonalcoholic fatty liver disease - a multisystem disease? World J Gastroenterol. (2016) 22:9488–505. 10.3748/wjg.v22.i43.9488 27920470 PMC5116593

[5] Selvakumar PKC Kabbany MN Nobili V Alkhouri N. Nonalcoholic fatty liver disease in children: hepatic and extrahepatic complications. Pediatr Clin North Am. (2017) 64:659–75. 10.1016/j.pcl.2017.01.008 28502444

[6] Reaven GM. Banting Lecture 1988. Role of insulin resistance in human disease. Diabetes. (1988) 37:1595–607. 3056758 10.2337/diab.37.12.1595

[7] Bussler S Penke M Flemming G Elhassan YS Kratzsch J Sergeyev E. Novel insights in the metabolic syndrome in childhood and adolescence. Horm Res Paediatr. (2017) 88:181–93. 10.1159/000479510 28848168

[8] Lonardo A Ballestri S Marchesini G Angulo P Loria P. Nonalcoholic fatty liver disease: a precursor of the metabolic syndrome. Dig Liver Dis. (2015) 47:181–90. 10.1016/j.dld.2014.09.020 25739820

[9] Asrih M Jornayvaz FR. Metabolic syndrome and nonalcoholic fatty liver disease: is insulin resistance the link? Mol Cell Endocrinol. (2015) 418(Pt 1):55–65. 10.1016/j.mce.2015.02.018 25724480

[10] Schwimmer JB Deutsch R Kahen T Lavine JE Stanley C Behling C. Prevalence of fatty liver in children and adolescents. Pediatrics. (2006) 118:1388–93. 10.1542/peds.2006-1212 17015527

[11] Mencin AA Lavine JE. Nonalcoholic fatty liver disease in children. Curr Opin Clin Nutr Metab Care. (2011) 14:151–7. 10.1097/MCO.0b013e328342baec 21178608

[12] Lawlor DA Callaway M Macdonald-Wallis C Anderson E Fraser A Howe LD. Nonalcoholic fatty liver disease, liver fibrosis, and cardiometabolic risk factors in adolescence: a cross-sectional study of 1874 general population adolescents. J Clin Endocrinol Metab. (2014) 99:E410–7. 10.1210/jc.2013-3612 24471572 PMC3955251

[13] Caserta CA Pendino GM Amante A Vacalebre C Fiorillo MT Surace P. Cardiovascular risk factors, nonalcoholic fatty liver disease, and carotid artery intima-media thickness in an adolescent population in southern Italy. Am J Epidemiol. (2010) 171:1195–202. 10.1093/aje/kwq073 20457571

[14] Yu EL Golshan S Harlow KE Angeles JE Durelle J Goyal NP. Prevalence of nonalcoholic fatty liver disease in children with obesity. J Pediatr. (2018) 207:64–70. 10.1016/j.jpeds.2018.11.021 30559024 PMC6440815

[15] Anderson EL Howe LD Jones HE Higgins JP Lawlor DA Fraser A. The prevalence of non-alcoholic fatty liver disease in children and adolescents: a systematic review and meta-analysis. PLoS ONE. (2015) 10:e0140908. 10.1371/journal.pone.0140908 26512983 PMC4626023

[16] Denzer C Thiere D Muche R Koenig W Mayer H Kratzer W. Gender-specific prevalences of fatty liver in obese children and adolescents: roles of body fat distribution, sex steroids, and insulin resistance. J Clin Endocrinol Metab. (2009) 94:3872–81. 10.1210/jc.2009-1125 19773396

[17] Sartorio A Del Col A Agosti F Mazzilli G Bellentani S Tiribelli C. Predictors of non-alcoholic fatty liver disease in obese children. Eur J Clin Nutr. (2007) 61:877–83. 10.1038/sj.ejcn.1602588 17151586

[18] Yoo J Lee S Kim K Yoo S Sung E Yim J. Relationship between insulin resistance and serum alanine aminotransferase as a surrogate of NAFLD (nonalcoholic fatty liver disease) in obese Korean children. Diabetes Res Clin Pract. (2008) 81:321–6. 10.1016/j.diabres.2008.05.006 18571268

[19] Fu JF Shi HB Liu LR Jiang P Liang L Wang CL. Non-alcoholic fatty liver disease: an early mediator predicting metabolic syndrome in obese children? World J Gastroenterol. (2011) 17:735–42. 10.3748/wjg.v17.i6.735 21390143 PMC3042651

[20] Mager DR Yap J Rodriguez-Dimitrescu C Mazurak V Ball G Gilmour S. Anthropometric measures of visceral and subcutaneous fat are important in the determination of metabolic dysregulation in boys and girls at risk for nonalcoholic fatty liver disease. Nutr Clin Pract. (2013) 28:101–11. 10.1177/0884533612454884 23042833

[21] Lee S Rivera-Vega M Alsayed HM Boesch C Libman I. Metabolic inflexibility and insulin resistance in obese adolescents with non-alcoholic fatty liver disease. Pediatr Diabetes. (2015) 16:211–8. 10.1111/pedi.12141 24754380 PMC4339626

[22] Boyraz M Hatipoglu N Sari E Akçay A Taşkin N Ulucan K. Non-alcoholic fatty liver disease in obese children and the relationship between metabolic syndrome criteria. Obes Res Clin Pract. (2014) 8:e356-63. 10.1016/j.orcp.2013.08.003 25091357

[23] Gupta R Bhangoo A Matthews NA Anhalt H Matta Y Lamichhane B. The prevalence of non-alcoholic fatty liver disease and metabolic syndrome in obese children. J Pediatr Endocrinol Metab. (2011) 24:907–11. 10.1515/JPEM.2011.282 22308841

[24] Love-Osborne KA Nadeau KJ Sheeder J Fenton LZ Zeitler P. Presence of the metabolic syndrome in obese adolescents predicts impaired glucose tolerance and nonalcoholic fatty liver disease. J Adolesc Health. (2008) 42:543–8. 10.1016/j.jadohealth.2007 18486862 PMC2601690

[25] El-Karaksy HM El-Raziky MS Fouad HM Anwar GM El-Mougy FM El-Koofy NM. The value of different insulin resistance indices in assessment of non-alcoholic fatty liver disease in overweight/obese children. Diabetes Metab Syndr. (2015) 9:114–9. 10.1016/j.dsx.2013.10.008 25470627

[26] Di Bonito P Sanguigno E Di Fraia T Forziato C Boccia G Saitta F. Association of elevated serum alanine aminotransferase with metabolic factors in obese children: sex-related analysis. Metabolism. (2009) 58:368–72. 10.1016/j.metabol.2008.10.010 19217453

[27] Shi HB Fu JF Liang L Wang CL Zhu JF Zhou F. Prevalence of nonalcoholic fatty liver disease and metabolic syndrome in obese children. Zhonghua Er Ke Za Zhi. (2009) 47:114–8. 19573457

[28] Lonardo A Nascimbeni F Ballestri S Fairweather D Win S Than TA. Sex differences in NAFLD: state of the art and identification of research gaps. Hepatology. (2019). 10.1002/hep.30626. [Epub ahead of print]. 30924946 PMC6766425

[29] Rocha R Cotrim HP Bitencourt AG Barbosa DB Santos AS Almeida Ade M. Nonalcoholic fatty liver disease in asymptomatic Brazilian adolescents. World J Gastroenterol. (2009) 15:473–7. 10.3748/wjg.15.473 19152453 PMC2653370

[30] Boza C Viscido G Salinas J Crovari F Funke R Perez G. Laparoscopic sleeve gastrectomy in obese adolescents: results in 51 patients. Surg Obes Relat Dis. (2012) 8:133–7; discussion 137–9. 10.1016/j.soard.2011.11.021 22433934

[31] Jain V Jana M Upadhyay B Ahmad N Jain O Upadhyay AD. Prevalence, clinical & biochemical correlates of non-alcoholic fatty liver disease in overweight adolescents. Indian J Med Res. (2018) 148:291–301. 10.4103/ijmr.IJMR_1966_16 30425219 PMC6251268

[32] Zhou X Hou DQ Duan JL Sun Y Cheng H Zhao XY. Prevalence of nonalcoholic fatty liver disease and metabolic abnormalities in 387 obese children and adolescents in Beijing, China. Zhonghua Liu Xing Bing Xue Za Zhi. (2013) 34:446–50. 10.3760/cma.j.issn.0254-6450.2013.05.008 24016432

[33] Mohanty SR Troy TN Huo D O'Brien BL Jensen DM Hart J. Influence of ethnicity on histological differences in non-alcoholic fatty liver disease. J Hepatol. (2009) 50:797–804. 10.1016/j.jhep.2008.11.017 19231016

[34] Williams CD Stengel J Asike MI Torres DM Shaw J Contreras M. Prevalence of nonalcoholic fatty liver disease and nonalcoholic steatohepatitis among a largely middle-aged population utilizing ultrasound and liver biopsy: a prospective study. Gastroenterology. (2011) 140:124–31. 10.1053/j.gastro.2010.09.038 20858492

[35] Guerrero R Vega GL Grundy SM Browning JD. Ethnic differences in hepatic steatosis: an insulin resistance paradox? Hepatology. (2009) 49:791–801. 10.1002/hep.22726 19105205 PMC2675577

[36] Kallwitz ER Kumar M Aggarwal R Berger R Layden-Almer J Gupta N. Ethnicity and nonalcoholic fatty liver disease in an obesity clinic: the impact of triglycerides. Dig Dis Sci. (2008) 53:1358–63. 10.1007/s10620-008-0234-x 18347982

[37] Tricò D Caprio S Rosaria Umano G Pierpont B Nouws J Galderisi A. Metabolic features of nonalcoholic fatty liver (NAFL) in obese adolescents: findings from a multiethnic cohort. Hepatology. (2018) 68:1376–90. 10.1002/hep.30035 29665034 PMC6173637

[38] Lomonaco R Ortiz-Lopez C Orsak B Finch J Webb A Bril F. Role of ethnicity in overweight and obese patients with nonalcoholic steatohepatitis. Hepatology. (2011) 54:837–45. 10.1002/hep.24483 21674556

[39] Franzese A Vajro P Argenziano A Puzziello A Iannucci MP Saviano MC. Liver involvement in obese children. Ultrasonography and liver enzyme levels at diagnosis and during follow-up in an Italian population. Dig Dis Sci. (1997) 42:1428–32. 9246041 10.1023/a:1018850223495

[40] Bedogni G Gastaldelli A Tiribelli C Agosti F De Col A Fessehatsion R. Relationship between glucose metabolism and non-alcoholic fatty liver disease severity in morbidly obese women. J Endocrinol Invest. (2014) 37:739–44. 10.1007/s40618-014-0101-x 24906975

[41] Pacifico L Poggiogalle E Cantisani V Menichini G Ricci P Ferraro F. Pediatric nonalcoholic fatty liver disease: a clinical and laboratory challenge. World J Hepatol. (2010) 2:275–88. 10.4254/wjh.v2.i7.275 21161009 PMC2998974

[42] Vos MB Abrams SH Barlow SE Caprio S Daniels SR Kohli R. NASPGHAN clinical practice guideline for the diagnosis and treatment of nonalcoholic fatty liver disease in children: recommendations from the expert committee on NAFLD (ECON) and the North American Society of Pediatric Gastroenterology, Hepatology and Nutrition (NASPGHAN). J Pediatr Gastroenterol Nutr. (2017) 64:319–34. 10.1097/MPG.0000000000001482 28107283 PMC5413933

[43] Lavine JE Schwimmer JB Molleston JP Scheimann AO Murray KF Abrams SH. Treatment of nonalcoholic fatty liver disease in children: TONIC trial design. Contemp Clin Trials. (2010) 31:62–70. 10.1016/j.cct.2009.09.001 19761871 PMC2936451

[44] Colantonio DA Kyriakopoulou L Chan MK Daly CH Brinc D Venner AA. Closing the gaps in pediatric laboratory reference intervals: a CALIPER database of 40 biochemical markers in a healthy and multiethnic population of children. Clin Chem. (2012) 58:854–68. 10.1373/clinchem.2011.177741 22371482

[45] Schwimmer JB Newton KP Awai HI Choi LJ Garcia MA Ellis LL. Paediatric gastroenterology evaluation of overweight and obese children referred from primary care for suspected non-alcoholic fatty liver disease. Aliment Pharmacol Ther. (2013) 38:1267–77. 10.1111/apt.12518 24117728 PMC3984047

[46] Bussler S Vogel M Pietzner D Harms K Buzek T Penke M. New pediatric percentiles of liver enzyme serum levels (alanine aminotransferase, aspartate aminotransferase, γ-glutamyltransferase): effects of age, sex, body mass index, and pubertal stage. Hepatology. (2018) 68:1319–30. 10.1002/hep.29542 28926121

[47] Zhang X Ji X Wang Q Li JZ. New insight into inter-organ crosstalk contributing to the pathogenesis of non-alcoholic fatty liver disease (NAFLD). Protein Cell. (2018) 9:164–77. 10.1007/s13238-017-0436-0 28643267 PMC5818366

[48] Fang YL Chen H Wang CL Liang L. Pathogenesis of non-alcoholic fatty liver disease in children and adolescence: from two hit theory to multiple hit model. World J Gastroenterol. (2018) 24:2974–83. 10.3748/wjg.v24.i27.2974 30038464 PMC6054950

[49] Clemente MG Mandato C Poeta M Vajro P. Pediatric non-alcoholic fatty liver disease: recent solutions, unresolved issues, and future research directions. World J Gastroenterol. (2016) 22:8078–93. 10.3748/wjg.v22.i36.8078 27688650 PMC5037077

[50] Wesolowski SR Kasmi KC Jonscher KR Friedman JE. Developmental origins of NAFLD: a womb with a clue. Nat Rev Gastroenterol Hepatol. (2017) 14:81–96. 10.1038/nrgastro.2016.160 27780972 PMC5725959

[51] Sookoian S Pirola CJ. Review article: shared disease mechanisms between non-alcoholic fatty liver disease and metabolic syndrome - translating knowledge from systems biology to the bedside. Aliment Pharmacol Ther. (2019) 49:516–27. 10.1111/apt.15163 30714632

[52] Valerio G Maffeis C Saggese G Ambruzzi MA Balsamo A Bellone S. Diagnosis, treatment and prevention of pediatric obesity: consensus position statement of the Italian Society for Pediatric Endocrinology and Diabetology and the Italian Society of Pediatrics. Ital J Pediatr. (2018) 44:88. 10.1186/s13052-018-0525-6 30064525 PMC6069785

[53] Mager DR Patterson C So S Rogenstein CD Wykes LJ Roberts EA. Dietary and physical activity patterns in children with fatty liver. Eur J Clin Nutr. (2010) 64:628–35. 10.1038/ejcn.2010.35 20216561

[54] Nobili V Manco M Devito R Di Ciommo V Comparcola D Sartorelli MR. Lifestyle intervention and antioxidant therapy in children with nonalcoholic fatty liver disease: a randomized, controlled trial. Hepatology. (2008) 48:119–28. 10.1002/hep.22336 18537181

[55] Pozzato C Verduci E Scaglioni S Radaelli G Salvioni M Rovere A. Liver fat change in obese children after a 1-year nutrition-behavior intervention. J Pediatr Gastroenterol Nutr. (2010) 51:331–5. 10.1097/MPG.0b013e3181d70468 20562718

[56] Balkau B Mhamdi L Oppert JM Nolan J Golay A Porcellati F. Physical activity and insulin sensitivity: the RISC study. Diabetes. (2008) 57:2613–8. 10.2337/db07-1605 18591396 PMC2551669

[57] Hatton G Alterio T Nobili V Mann JP. Unmet needs in pediatric NAFLD research: what do we need to prioritize for the future? Expert Rev Gastroenterol Hepatol. (2018) 12:961–7. 10.1080/17474124.2018.1512853 30117754

[58] Mann JP Raponi M Nobili V. Clinical implications of understanding the association between oxidative stress and pediatric NAFLD. Expert Rev Gastroenterol Hepatol. (2017) 11:371–82. 10.1080/17474124.2017.1291340 28162008

[59] Clemente MG Mandato C Poeta M Vajro P. Pediatric non-alcoholic fatty liver disease: recent solutions, unresolved issues, and future research directions. World J Gastroenterol. (2016) 22:8078–93. 10.3748/wjg.v22.i36.8078 27688650 PMC5037077

[60] Cai D Yuan M Frantz DF Melendez PA Hansen L Lee J. Local and systemic insulin resistance resulting from hepatic activation of IKK-beta and NF-kappaB. Nat Med. (2005) 11:183–90. 10.1038/nm1166 15685173 PMC1440292

[61] Sumida Y Niki E Naito Y Yoshikawa T. Involvement of free radicals and oxidative stress in NAFLD/NASH. Free Radic Res. (2013) 47:869–80. 10.3109/10715762.2013.837577 24004441

[62] Pirgon Ö Bilgin H Çekmez F Kurku H Dündar BN. Association between insulin resistance and oxidative stress parameters in obese adolescents with non-alcoholic fatty liver disease. J Clin Res Pediatr Endocrinol. (2013) 5:33–9. 10.4274/Jcrpe.825 23367495 PMC3628390

[63] Ashraf NU Sheikh TA. Endoplasmic reticulum stress and oxidative stress in the pathogenesis of non-alcoholic fatty liver disease. Free Radic Res. (2015) 49:1405–18. 10.3109/10715762.2015.1078461 26223319

[64] Nassir F Rector RS Hammoud GM Ibdah JA. Pathogenesis and prevention of hepatic steatosis. Gastroenterol Hepatol. (2015) 11:167–75. 27099587 PMC4836586

[65] Paradies G Paradies V Ruggiero FM Petrosillo G. Oxidative stress, cardiolipin and mitochondrial dysfunction in nonalcoholic fatty liver disease. World J Gastroenterol. (2014) 20:14205–18. 10.3748/wjg.v20.i39.14205 25339807 PMC4202349

[66] Vacca M Allison M Griffin JL Vidal-Puig A. Fatty acid and glucose sensors in hepatic lipid metabolism: implications in NAFLD. Semin Liver Dis. (2015) 35:250–61. 10.1055/s-0035-1562945 26378642

[67] Leung DH Yimlamai D. The intestinal microbiome and paediatric liver disease. Lancet Gastroenterol Hepatol. (2017) 2:446–55. 10.1016/S2468-1253(16)30241-2 28497760

[68] Boursier J Diehl AM. Implication of gut microbiota in nonalcoholic fatty liver disease. PLoS Pathog. (2015) 11:e1004559. 10.1371/journal.ppat.1004559 25625278 PMC4308105

[69] Doulberis M Kotronis G Gialamprinou D Kountouras J Katsinelos P. Non-alcoholic fatty liver disease: an update with special focus on the role of gut microbiota. Metabolism. (2017) 71:182–97. 10.1016/j.metabol.2017.03.013 28521872

[70] Alisi A Manco M Devito R Piemonte F Nobili V. Endotoxin and plasminogen activator inhibitor-1 serum levels associated with nonalcoholic steatohepatitis in children. J Pediatr Gastroenterol Nutr. (2010) 50:645–9. 10.1097/MPG.0b013e3181c7bdf1 20400911

[71] Ceccarelli S Panera N Mina M Gnani D De Stefanis C Crudele A. LPS-induced TNF-α factor mediates pro-inflammatory and pro-fibrogenic pattern in non-alcoholic fatty liver disease. Oncotarget. (2015) 6:41434–52. 10.18632/oncotarget.5163 26573228 PMC4747165

[72] Del Chierico F Nobili V Vernocchi P Russo A Stefanis C Gnani D. Gut microbiota profiling of pediatric nonalcoholic fatty liver disease and obese patients unveiled by an integrated meta-omics-based approach. Hepatology. (2017) 65:451–64. 10.1002/hep.28572 27028797

[73] Belei O Olariu L Dobrescu A Marcovici T Marginean O. The relationship between non-alcoholic fatty liver disease and small intestinal bacterial overgrowth among overweight and obese children and adolescents. J Pediatr Endocrinol Metab. (2017) 30:1161–8. 10.1515/jpem-2017-0252 28988228

[74] Zhu L Baker SS Gill C Liu W Alkhouri R Baker RD. Characterization of gut microbiomes in nonalcoholic steatohepatitis (NASH) patients: a connection between endogenous alcohol and NASH. Hepatology. (2013) 57:601–9. 10.1002/hep.26093 23055155

[75] Pierri L Saggese P Guercio Nuzio S Troisi J Di Stasi M Poeta M. Relations of gut liver axis components and gut microbiota in obese children with fatty liver: A pilot study. Clin Res Hepatol Gastroenterol. (2018) 42:387–90. 10.1016/j.clinre.2018.03.015 29773420

[76] D'Adamo E Santoro N Caprio S. Metabolic syndrome in pediatrics: old concepts revised, new concepts discussed. Curr Probl Pediatr Adolesc Health Care. (2013) 43:114–23. 10.1016/j.cppeds.2013.02.004 23582593

[77] Lin X Lim IY Wu Y Teh AL Chen L Aris IM. Developmental pathways to adiposity begin before birth and are influenced by genotype, prenatal environment and epigenome. BMC Med. (2017) 15:50. 10.1186/s12916-017-0800-1 28264723 PMC5340003

[78] D'Adamo E Cali AM Weiss R Santoro N Pierpont B Northrup V. Central role of fatty liver in the pathogenesis of insulin resistance in obese adolescents. Diabetes Care. (2010) 33:1817–22. 10.2337/dc10-0284 20668154 PMC2909068

[79] Patton HM Yates K Unalp-Arida A Behling CA Huang TTK Rosenthal P. Association between metabolic syndrome and liver histology among children with nonalcoholic fatty liver disease. Am J Gastroenterol. (2010) 105:2093–102. 10.1038/ajg.2010.152 20372110 PMC3070291

[80] Rehm JL Connor EL Wolfgram P Eickhoff JC Reeder SB Allen DB. Predicting hepatic steatosis in a racially and ethnically diverse cohort of adolescent girls. J Pediatr. (2014) 165:319–25.e1. 10.1016/j.jpeds.2014.04.019 24857521 PMC4131842

[81] Prokopowicz Z Malecka-Tendera E Matusik P. Predictive value of adiposity level, metabolic syndrome, and insulin resistance for the risk of nonalcoholic fatty liver disease diagnosis in obese children. Can J Gastroenterol Hepatol. (2018) 2018:9465784. 10.1155/2018/9465784 29854716 PMC5944281

[82] Hampe CS Shaffer ML Roth CL. Associations between liver enzyme levels and parameters of the metabolic syndrome in obese children. Horm Res Paediatr. (2017) 88:265–73. 10.1159/000479868 28898874

[83] Kim G Giannini C Pierpont B Feldstein AE Santoro N Kursawe R. Longitudinal effects of MRI-measured hepatic steatosis on biomarkers of glucose homeostasis and hepatic apoptosis in obese youth. Diabetes Care. (2013) 36:130–6. 10.2337/dc12-0277 22933439 PMC3526202

[84] Caprio S Pierpont B Kursawe R. The adipose tissue expandability hypothesis: a potential mechanism for insulin resistance in obese youth. Horm Mol Biol Clin Investig. (2018) 33:2. 10.1515/hmbci-2018-0005 29596053

[85] Caprio S Perry R Kursawe R. Adolescent obesity and insulin resistance: roles of ectopic fat accumulation and adipose inflammation. Gastroenterology. (2017) 152:1638–46. 10.1053/j.gastro.2016.12.051 28192105 PMC9390070

[86] Taksali SE Caprio S Dziura J Dufour S Calí AM Goodman TR. High visceral and low abdominal subcutaneous fat stores in the obese adolescent: a determinant of an adverse metabolic phenotype. Diabetes. (2008) 57:367–71. 10.2337/db07-0932 17977954

[87] Cali AM Caprio S. Ectopic fat deposition and the metabolic syndrome in obese children and adolescents. Horm Res. (2009) 71(Suppl. 1):2–7. 10.1159/000178028 19153496

[88] Umano GR Shabanova V Pierpont B Mata M Nouws J Tricò D. A low visceral fat proportion, independent of total body fat mass, protects obese adolescent girls against fatty liver and glucose dysregulation: a longitudinal study. Int J Obes. (2018) 43:673–82. 10.1038/s41366-018-0227-6 30337653 PMC9354568

[89] Kursawe R Eszlinger M Narayan D Liu T Bazuine M Cali AM. Cellularity and adipogenic profile of the abdominal subcutaneous adipose tissue from obese adolescents: association with insulin resistance and hepatic steatosis. Diabetes. (2010) 59:2288–96. 10.2337/db10-0113 20805387 PMC2927952

[90] Kursawe R Caprio S Giannini C Narayan D Lin A D'Adamo E. Decreased transcription of ChREBP-α/β isoforms in abdominal subcutaneous adipose tissue of obese adolescents with prediabetes or early type 2 diabetes: associations with insulin resistance and hyperglycemia. Diabetes. (2013) 62:837–44. 10.2337/db12-0889 23209190 PMC3581226

[91] Amor AJ Perea V. Dyslipidemia in nonalcoholic fatty liver disease. Curr Opin Endocrinol Diabetes Obes. (2019) 26:103–8. 10.1097/MED.0000000000000464 30694825

[92] Mahaling DU Basavaraj MM Bika AJ Comparison of lipid profile in different grades of non-alcoholic fatty liver disease diagnosed on ultrasound. Asian Pac J Trop Biomed. (2013) 3:907–12. 10.1016/S2221-1691(13)60177-X

[93] Peng K Mo Z Tian G. Serum lipid abnormalities and nonalcoholic fatty liver disease in adult males. Am J Med Sci. (2017) 353:236–41. 10.1016/j.amjms.2017.01.002 28262209

[94] Zheng J Zhou Y Zhang K Qi Y An S Wang S. Association between nonalcoholic fatty liver disease and subclinical atherosclerosis: a cross-sectional study on population over 40 years old. BMC Cardiovasc Disord. (2018) 18:147. 10.1186/s12872-018-0877-2 30012085 PMC6048911

[95] Deeb A Attia S Mahmoud S Elhaj G Elfatih A. Dyslipidemia and fatty liver disease in overweight and obese children. J Obes. (2018) 2018:8626818. 10.1155/2018/8626818 30009050 PMC6020453

[96] Nguyen D Kit B Carroll M. Abnormal Cholesterol Among Children and Adolescents in the United States, 2011-2014. NCHS Data Brief (2015):1–8. 26727279

[97] Montali A Truglio G Martino F Ceci F Ferraguti G Ciociola E Atherogenic dyslipidemia in children: evaluation of clinical, biochemical and genetic aspects. PLoS ONE. (2015) 10:e0120099 10.1371/journal.pone.0120099 25897955 PMC4405441

[98] Nobili V Alkhouri N Bartuli A Manco M Lopez R Alisi A. Severity of liver injury and atherogenic lipid profile in children with nonalcoholic fatty liver disease. Pediatr Res. (2010) 67:665–70. 10.1203/PDR.0b013e3181da4798 20496475

[99] McLaughlin T Reaven G Abbasi F Lamendola C Saad M Waters D. Is there a simple way to identify insulin-resistant individuals at increased risk of cardiovascular disease? Am J Cardiol. (2005) 96:399–404. 10.1016/j.amjcard.2005.03.085 16054467

[100] Quijada Z Paoli M Zerpa Y Camacho N Cichetti R Villarroel V. The triglyceride/HDL-cholesterol ratio as a marker of cardiovascular risk in obese children; association with traditional and emergent risk factors. Pediatr Diabetes. (2008) 9:464–71. 10.1111/j.1399-5448.2008.00406.x 18507788

[101] Corey KE Vuppalanchi R Vos M Kohli R Molleston JP Wilson L Unalp-Arida A Cummings OW. Improvement in liver histology is associated with reduction in dyslipidemia in children with nonalcoholic fatty liver disease. J Pediatr Gastroenterol Nutr. (2015) 60:360–7. 10.1097/MPG.0000000000000584 25714579 PMC4341955

[102] Pacifico L Bonci E Andreoli G Romaggioli S Di Miscio R Lombardo CV. Association of serum triglyceride-to-HDL cholesterol ratio with carotid artery intima-media thickness, insulin resistance and nonalcoholic fatty liver disease in children and adolescents. Nutr Metab Cardiovasc Dis. (2014) 24:737–43. 10.1016/j.numecd.2014.01.010 24656140

[103] Di Bonito P Valerio G Grugni G Licenziati MR Maffeis C Manco M. Comparison of non-HDL-cholesterol versus triglycerides-to-HDL-cholesterol ratio in relation to cardiometabolic risk factors and preclinical organ damage in overweight/obese children: the CARITALY study. Nutr Metab Cardiovasc Dis. (2015) 25:489–94. 10.1016/j.numecd.2015.01.012 25813687

[104] Bălănescu A Bălănescu P Comănici V Stan I Acs B1 Prisăcariu L. Lipid profile pattern in pediatric overweight population with or without NAFLD in relation to IDF criteria for metabolic syndrome: a preliminary study. Rom J Intern Med. (2018) 56:47–54. 10.1515/rjim-2017-0040 29080393

[105] Dowla S Aslibekyan S Goss A Fontaine K Ashraf AP. Dyslipidemia is associated with pediatric nonalcoholic fatty liver disease. J Clin Lipidol. (2018) 12:981–7. 10.1016/j.jacl.2018.03.089 29699915 PMC8513128

[106] Jimenez-Rivera C Hadjiyannakis S Davila J Hurteau J Aglipay M Barrowman N. Prevalence and risk factors for non-alcoholic fatty liver in children and youth with obesity. BMC Pediatr. (2017) 17:113. 10.1186/s12887-017-0867-z 28446162 PMC5406891

[107] Lee JH Jeong SJ. What is the appropriate strategy for diagnosing NAFLD using ultrasonography in obese children? World J Pediatr. (2017) 13:248–54. 10.1007/s12519-017-0008-7 28101773

[108] Iqbal AM Dahl AR Lteif A Kumar S. Vitamin D deficiency: a potential modifiable risk factor for cardiovascular disease in children with severe obesity. Children (Basel). (2017) 4:E80. 10.3390/children4090080 28846662 PMC5615270

[109] Kumar J Muntner P Kaskel FJ Hailpern SM Melamed ML. Prevalence and associations of 25-hydroxyvitamin D deficiency in US children: NHANES 2001-2004. Pediatrics. (2009) 124:e362-70. 10.1542/peds.2009-0051 19661054 PMC3749840

[110] Turer CB Lin H Flores G. Prevalence of vitamin D deficiency among overweight and obese US children. Pediatrics. (2013) 131:e152-61. 10.1542/peds.2012-1711 23266927

[111] Olson ML Maalouf NM Oden JD White PC Hutchison MR. Vitamin D deficiency in obese children and its relationship to glucose homeostasis. J Clin Endocrinol Metab. (2012) 97:279–85. 10.1210/jc.2011-1507 22072738 PMC3251943

[112] Misra M Pacaud D Petryk A Collett-Solberg PF Kappy M Lawson Wilkins Pediat Endocrine S. Vitamin D deficiency in children and its management: review of current knowledge and recommendations. Pediatrics. (2008) 122:398–417. 10.1542/peds.2007-1894 18676559

[113] Eliades M Spyrou E Agrawal N Lazo M Brancati FL Potter JJ. Meta-analysis: vitamin D and non-alcoholic fatty liver disease. Aliment Pharmacol Ther. (2013) 38:246–54. 10.1111/apt.12377 23786213

[114] Targher G Bertolini L Scala L Cigolini M Zenari L Falezza G. Associations between serum 25-hydroxyvitamin D3 concentrations and liver histology in patients with non-alcoholic fatty liver disease. Nutr Metab Cardiovasc Dis. (2007) 17:517–24. 10.1016/j.numecd.2006.04.002 16928437

[115] Manco M Ciampalini P Nobili V. Low levels of 25-hydroxyvitamin D(3) in children with biopsy-proven nonalcoholic fatty liver disease. Hepatology. (2010) 51:2229. 10.1002/hep.23724 20513013

[116] Nobili V Giorgio V Liccardo D Bedogni G Morino G Alisi A. Vitamin D levels and liver histological alterations in children with nonalcoholic fatty liver disease. Eur J Endocrinol. (2014) 170:547–53. 10.1530/EJE-13-0609 24412930

[117] Black LJ Jacoby P She Ping-Delfos WC Mori TA Beilin LJ Olynyk JK. Low serum 25-hydroxyvitamin D concentrations associate with non-alcoholic fatty liver disease in adolescents independent of adiposity. J Gastroenterol Hepatol. (2014) 29:1215–22. 10.1111/jgh.12541 24611991

[118] Luger M Kruschitz R Kienbacher C Traussnigg S Langer FB Schindler K. Prevalence of liver fibrosis and its association with non-invasive fibrosis and metabolic markers in morbidly obese patients with vitamin D deficiency. Obes Surg. (2016) 26:2425–32. 10.1007/s11695-016-2123-2 26989059 PMC5018030

[119] Roth CL Elfers CT Figlewicz DP Melhorn SJ Morton GJ Hoofnagle A. Vitamin D deficiency in obese rats exacerbates nonalcoholic fatty liver disease and increases hepatic resistin and Toll-like receptor activation. Hepatology. (2012) 55:1103–11. 10.1002/hep.24737 21994008

[120] Kwok RM Torres DM Harrison SA. Vitamin D and NAFLD: is it more than just an association? Hepatology. (2013) 58:1166–74. 10.1002/hep.26390 23504808

[121] Wang TJ Zhang F Richards JB Kestenbaum B van Meurs JB Berry D. Common genetic determinants of vitamin D insufficiency: a genome-wide association study. Lancet. (2010) 376:180–8. 10.1016/S0140-6736(10)60588-0 20541252 PMC3086761

[122] Lu L Sheng H Li H Gan W Liu C Zhu J. Associations between common variants in GC and DHCR7/NADSYN1 and vitamin D concentration in Chinese Hans. Hum Genet. (2012) 131:505–12. 10.1007/s00439-011-1099-1 21972121

[123] Gibson PS Quaglia A Dhawan A Wu H Lanham-New S Hart KH. Vitamin D status and associated genetic polymorphisms in a cohort of UK children with non-alcoholic fatty liver disease. Pediatr Obes. (2018) 13:433–41. 10.1111/ijpo.12293 29761652 PMC6032876

[124] Pyrzak B Witkowska-Sedek E Krajewska M Demkow U Kucharska AM. Metabolic and immunological consequences of vitamin D deficiency in obese children. Adv Exp Med Biol. (2015) 840:13–9. 10.1007/5584_2014_81 25315624

[125] Ganji V Zhang X Shaikh N Tangpricha V. Serum 25-hydroxyvitamin D concentrations are associated with prevalence of metabolic syndrome and various cardiometabolic risk factors in US children and adolescents based on assay-adjusted serum 25-hydroxyvitamin D data from NHANES 2001-2006. Am J Clin Nutr. (2011) 94:225–33. 10.3945/ajcn.111.013516 21613551

[126] Lee SH Kim SM Park HS Choi KM Cho GJ Ko BJ. Serum 25-hydroxyvitamin D levels, obesity and the metabolic syndrome among Korean children. Nutr Metab Cardiovasc Dis. (2013) 23:785–91. 10.1016/j.numecd.2012.04.013 22762845

[127] Pacifico L Anania C Osborn JF Ferraro F Bonci E Olivero E. Low 25(OH)D3 levels are associated with total adiposity, metabolic syndrome, and hypertension in Caucasian children and adolescents. Eur J Endocrinol. (2011) 165:603–11. 10.1530/EJE-11-0545 21753070

[128] Janczyk W Socha P Lebensztejn D Wierzbicka A Mazur A Neuhoff-Murawska J. Omega-3 fatty acids for treatment of non-alcoholic fatty liver disease: design and rationale of randomized controlled trial. BMC Pediatr. (2013) 13:85. 10.1186/1471-2431-13-85 23702094 PMC3672084

[129] Nobili V Alisi A Musso G Scorletti E Calder PC Byrne CD. Omega-3 fatty acids: mechanisms of benefit and therapeutic effects in pediatric and adult NAFLD. Crit Rev Clin Lab Sci. (2016) 53:106–20. 10.3109/10408363.2015.1092106 26463349

[130] Della Corte C Carpino G De Vito R De Stefanis C Alisi A Cianfarani S. Docosahexanoic acid plus vitamin D treatment improves features of NAFLD in children with serum vitamin D deficiency: results from a single centre trial. PLoS ONE. (2016) 11:e0168216. 10.1371/journal.pone.0168216 27977757 PMC5158039

[131] Parola M Vajro P. Nocturnal hypoxia in obese-related obstructive sleep apnea as a putative trigger of oxidative stress in pediatric NAFLD progression. J Hepatol. (2016) 65:470–2. 10.1016/j.jhep.2016.05.042 27501737

[132] Sundaram SS Halbower A Pan Z Robbins K Capocelli KE Klawitte J. Nocturnal hypoxia induced oxidative stress promotes progression of pediatric non-alcoholic fatty liver disease. J Hepatol. (2016) 65:560–9. 10.1016/j.jhep.2016.04.010 27501738 PMC4992457

[133] Paschetta E Belci P Alisi A Liccardo D Cutrera R Musso G. OSAS-related inflammatory mechanisms of liver injury in nonalcoholic fatty liver disease. Mediat Inflamm. (2015) 2015:815721. 10.1155/2015/815721 25873773 PMC4383458

[134] Rankin EB Rha J Selak MA Unger TL Keith B Liu Q. Hypoxia-inducible factor 2 regulates hepatic lipid metabolism. Mol Cell Biol. (2009) 29:4527–38. 10.1128/MCB.00200-09 19528226 PMC2725738

[135] Nobili V Cutrera R Liccardo D Pavone M Devito R Giorgio V. Obstructive sleep apnea syndrome affects liver histology and inflammatory cell activation in pediatric nonalcoholic fatty liver disease, regardless of obesity/insulin resistance. Am J Respir Crit Care Med. (2014) 189:66–76. 10.1164/rccm.201307-1339OC 24256086

[136] Zhou Y Wei F Fan Y. High serum uric acid and risk of nonalcoholic fatty liver disease: a systematic review and meta-analysis. Clin Biochem. (2016) 49:636–42. 10.1016/j.clinbiochem.2015.12.010 26738417

[137] Richette P Bardin T. Gout. Lancet. (2010) 375:318–28. 10.1016/S0140-6736(09)60883-7 19692116

[138] Lonardo A Loria P Leonardi F Borsatti A Neri P Pulvirenti M Fasting insulin and uric acid levels but not indices of iron metabolism are independent predictors of non-alcoholic fatty liver disease. A case-control study. Dig Liver Dis. (2002) 34:204–11. 10.1016/S1590-8658(02)80194-3 11990393

[139] Yang C Yang S Xu W Zhang J Fu W Feng C. Association between the hyperuricemia and nonalcoholic fatty liver disease risk in a Chinese population: a retrospective cohort study. PLoS ONE. (2017) 12:e0177249. 10.1371/journal.pone.0177249 28510581 PMC5433681

[140] Petta S Cammà C Cabibi D Di Marco V Craxì A. Hyperuricemia is associated with histological liver damage in patients with non-alcoholic fatty liver disease. Aliment Pharmacol Ther. (2011) 34:757–66. 10.1111/j.1365-2036.2011.04788.x 21790685

[141] Feig DI Kang DH Johnson RJ. Uric acid and cardiovascular risk. N Engl J Med. (2008) 359:1811–21. 10.1056/NEJMra0800885 18946066 PMC2684330

[142] Edwards NL. The role of hyperuricemia in vascular disorders. Curr Opin Rheumatol. (2009) 21:132–7. 10.1097/BOR.0b013e3283257b96 19339923

[143] Zhu Y Hu Y Huang T Zhang Y Li Z Luo C. High uric acid directly inhibits insulin signalling and induces insulin resistance. Biochem Biophys Res Commun. (2014) 447:707–14. 10.1016/j.bbrc.2014.04.080 24769205

[144] Vandanmagsar B Youm YH Ravussin A Galgani JE Stadler K Mynatt RL. The NLRP3 inflammasome instigates obesity-induced inflammation and insulin resistance. Nat Med. (2011) 17:179–88. 10.1038/nm.2279 21217695 PMC3076025

[145] Hwang IC Suh SY Suh AR Ahn HY. The relationship between normal serum uric acid and nonalcoholic fatty liver disease. J Korean Med Sci. (2011) 26:386–91. 10.3346/jkms.2011.26.3.386 21394307 PMC3051086

[146] Wu SJ Zhu GQ Ye BZ Kong FQ Zheng ZX Zou H. Association between sex-specific serum uric acid and non-alcoholic fatty liver disease in Chinese adults: a large population-based study. Medicine (Baltimore). (2015) 94:e802. 10.1097/MD.0000000000000802 25929934 PMC4603030

[147] Vos MB Colvin R Belt P Molleston JP Murray KF Rosenthal P. Correlation of vitamin E, uric acid, and diet composition with histologic features of pediatric NAFLD. J Pediatr Gastroenterol Nutr. (2012) 54:90–6. 10.1097/MPG.0b013e318229da1a 22197855 PMC3208079

[148] Nobili V Mosca A De Vito R Raponi M Scorletti E Byrne CD. Liver zonation in children with non-alcoholic fatty liver disease: associations with dietary fructose and uric acid concentrations. Liver Int. (2018) 38:1102–9. 10.1111/liv.13661 29222961

[149] Sullivan JS Le MT Pan Z Rivard C Love-Osborne K Robbins K. Oral fructose absorption in obese children with non-alcoholic fatty liver disease. Pediatr Obes. (2015) 10:188–95. 10.1111/ijpo.238 24961681 PMC4948988

[150] Maersk M Belza A Stødkilde-Jørgensen H Ringgaard S Chabanova E Thomsen H. Sucrose-sweetened beverages increase fat storage in the liver, muscle, and visceral fat depot: a 6-mo randomized intervention study. Am J Clin Nutr. (2012) 95:283–9. 10.3945/ajcn.111.022533 22205311

[151] Lombardi R Pisano G Fargion S. Role of serum uric acid and ferritin in the development and progression of NAFLD. Int J Mol Sci. (2016) 17:548. 10.3390/ijms17040548 27077854 PMC4849004

[152] Mosca A Nobili V De Vito R Crudele A Scorletti E Villani A. Serum uric acid concentrations and fructose consumption are independently associated with NASH in children and adolescents. J Hepatol. (2017) 66:1031–6. 10.1016/j.jhep.2016.12.025 28214020

[153] European Workshop on the Impact of Endocrine Disrupters on Human Health and Wild life Weybridge: UK.2-4/ (1996).

[154] Treviño LS Katz TA. Endocrine disruptors and developmental origins of nonalcoholic fatty liver disease. Endocrinology. (2018) 159:20–31. 10.1210/en.2017-00887 29126168 PMC5761605

[155] Attia AM El-Banna SG Nomeir FR Abd El-Basser MI. Lindane-induced biochemical perturbations in rat serum and attenuation by omega-3 and Nigella sativa seed oil. Indian J Biochem Biophys. (2011) 48:184–90. 21793310

[156] Angrish MM Dominici CY Zacharewski TR. TCDD-elicited effects on liver, serum, and adipose lipid composition in C57BL/6 mice. Toxicol Sci. (2013) 131:108–15. 10.1093/toxsci/kfs277 22977169 PMC3537129

[157] Desai M Jellyman JK Ross MG. Epigenomics, gestational programming and risk of metabolic syndrome. Int J Obes (Lond). (2015) 39:633–41. 10.1038/ijo.2015.13 25640766

[158] Janesick A Blumberg B. Obesogens, stem cells and the developmental programming of obesity. Int J Androl. (2012) 35:437–48. 10.1111/j.1365-2605.2012.01247.x 22372658 PMC3358413

[159] Alonso-Magdalena P Vieira E Soriano S Menes L Burks D Quesada I. Bisphenol A exposure during pregnancy disrupts glucose homeostasis in mothers and adult male offspring. Environ Health Perspect. (2010) 118:1243–50. 10.1289/ehp.1001993 20488778 PMC2944084

[160] Ye X Wong LY Kramer J Zhou X Jia T Calafat AM. Urinary concentrations of bisphenol A and three other bisphenols in convenience samples of U.S. Adults during 2000-2014. Environ Sci Technol. (2015) 49:11834–9. 10.1021/acs.est.5b02135 26360019 PMC7948051

[161] Wei J Lin Y Li Y Ying C Chen J Song L. Perinatal exposure to bisphenol A at reference dose predisposes offspring to metabolic syndrome in adult rats on a high-fat diet. Endocrinology. (2011) 152:3049–61. 10.1210/en.2011-0045 21586551

[162] Menale C Grandone A Nicolucci C Cirillo G Crispi S Di Sessa A. Bisphenol A is associated with insulin resistance and modulates adiponectin and resistin gene expression in obese children. Pediatr Obes. (2017) 12:380–7. 10.1111/ijpo.12154 27187765

[163] Wei J Sun X Chen Y Li Y Song L Zhou Z. Perinatal exposure to bisphenol A exacerbates nonalcoholic steatohepatitis-like phenotype in male rat offspring fed on a high-fat diet. J Endocrinol. (2014) 222:313–25. 10.1530/JOE-14-0356 25112833

[164] Khalil N Ebert JR Wang L Belcher S Lee M Czerwinski SA. Bisphenol A and cardiometabolic risk factors in obese children. Sci Total Environ. (2014) 470-471:726–32. 10.1016/j.scitotenv.2013.09.088 24184549

[165] Verstraete SG Wojcicki JM Perito ER Rosenthal P. Bisphenol a increases risk for presumed non-alcoholic fatty liver disease in Hispanic adolescents in NHANES 2003-2010. Environ Health. (2018) 17:12. 10.1186/s12940-018-0356-3 29391015 PMC5796302

[166] Santoro N Kursawe R D'Adamo E Dykas DJ Zhang CK Bale AE. A common variant in the patatin-like phospholipase 3 gene (PNPLA3) is associated with fatty liver disease in obese children and adolescents. Hepatology. (2010) 52:1281–90. 10.1002/hep.23832 20803499 PMC3221304

[167] Marzuillo P Miraglia del Giudice E Santoro N. Pediatric fatty liver disease: role of ethnicity and genetics. World J Gastroenterol. (2014) 20:7347–55. 10.3748/wjg.v20.i23.7347 24966605 PMC4064080

[168] Jia JJ Zhang X Ge CR Jois M. The polymorphisms of UCP2 and UCP3 genes associated with fat metabolism, obesity and diabetes. Obes Rev. (2009) 10:519–26. 10.1111/j.1467-789X.2009.00569.x 19413708

[169] Kozlitina J Smagris E Stender S Nordestgaard BG Zhou HH Tybjærg-Hansen A. Exome-wide association study identifies a TM6SF2 variant that confers susceptibility to nonalcoholic fatty liver disease. Nat Genet. (2014) 46:352–6. 10.1038/ng.2901 24531328 PMC3969786

[170] Gijón MA Riekhof WR Zarini S Murphy RC Voelker DR. Lysophospholipid acyltransferases and arachidonate recycling in human neutrophils. J Biol Chem. (2008) 283:30235–45. 10.1074/jbc.M806194200 18772128 PMC2573059

[171] Lin YC Chang PF Chang MH Ni YH. A common variant in the peroxisome proliferator-activated receptor-γ coactivator-1α gene is associated with nonalcoholic fatty liver disease in obese children. Am J Clin Nutr. (2013) 97:326–31. 10.3945/ajcn.112.046417 23269818

[172] Dai D Wen F Zhou S Su Z Liu G2 Wang M. Association of MTTP gene variants with pediatric NAFLD: a candidate-gene-based analysis of single nucleotide variations in obese children. PLoS ONE. (2017) 12:e0185396. 10.1371/journal.pone.0185396 28953935 PMC5617203

[173] Mangge H Baumgartner BG Zelzer S Prüller F Schnedl WJ Reininghaus EZ. Patatin-like phospholipase 3 (rs738409) gene polymorphism is associated with increased liver enzymes in obese adolescents and metabolic syndrome in all ages. Aliment Pharmacol Ther. (2015) 42:99–105. 10.1111/apt.13232 25939720

[174] Browning JD Cohen JC Hobbs HH PNPLA3 and the pathogenesis and progression of pediatric NAFLD. Hepatology. (2010) 52:1189–92. 10.1002/hep.23946 20879029 PMC3135009

[175] Kumari M Schoiswohl G Chitraju C Paar M Cornaciu I Rangrez AY. Adiponutrin functions as a nutritionally regulated lysophosphatidic acid acyltransferase. Cell Metab. (2012) 15:691–702. 10.1016/j.cmet.2012.04.008 22560221 PMC3361708

[176] Perttilä J Huaman-Samanez C Caron S Tanhuanpää K Staels B Yki-Järvinen H. PNPLA3 is regulated by glucose in human hepatocytes, and its I148M mutant slows down triglyceride hydrolysis. Am J Physiol Endocrinol Metab. (2012) 302:E1063–9. 10.1152/ajpendo.00125.2011 22338072

[177] Yki-Järvinen H. Non-alcoholic fatty liver disease as a cause and a consequence of metabolic syndrome. Lancet Diabetes Endocrinol. (2014) 2:901–10. 10.1016/S2213-8587(14)70032-4 24731669

[178] Romeo S Kozlitina J Xing C Pertsemlidis A Cox D Pennacchio LA. Genetic variation in PNPLA3 confers susceptibility to nonalcoholic fatty liver disease. Nat Genet. (2008) 40:1461–5. 10.1038/ng.257 18820647 PMC2597056

[179] Romeo S Huang-Doran I Baroni MG Kotronen A. Unravelling the pathogenesis of fatty liver disease: patatin-like phospholipase domain-containing 3 protein. Curr Opin Lipidol. (2010) 21:247–52. 10.1097/MOL.0b013e328338ca61 20480550

[180] Santoro N Zhang CK Zhao H Pakstis AJ Kim G Kursawe R. A variant in the glucokinase regulatory protein (GCKR) gene is associated with fatty liver in obese children and adolescents. Hepatology. (2012) 55:781–9. 10.1002/hep.24806 22105854 PMC3288435

[181] Lin YC Chang PF Chang MH Ni YH. Genetic variants in GCKR and PNPLA3 confer susceptibility to nonalcoholic fatty liver disease in obese individuals. Am J Clin Nutr. (2014) 99:869–74. 10.3945/ajcn.113.079749 24477042

[182] Hudert CA Selinski S Rudolph B Bläker H Loddenkemper C Thielhorn R. Genetic determinants of steatosis and fibrosis progression in paediatric non-alcoholic fatty liver disease. Liver Int. (2019) 39:540–56. 10.1111/liv.14006 30444569

[183] Xu YP Liang L Wang CL Fu JF Liu PN Lv LQ. Association between UCP3 gene polymorphisms and nonalcoholic fatty liver disease in Chinese children. World J Gastroenterol. (2013) 19:5897–903. 10.3748/wjg.v19.i35.5897 24124336 PMC3793144

[184] Grandone A Cozzolino D Marzuillo P Cirillo G Di Sessa A Ruggiero L. TM6SF2 Glu167Lys polymorphism is associated with low levels of LDL-cholesterol and increased liver injury in obese children. Pediatr Obes. (2016) 11:115–9. 10.1111/ijpo.12032 25893821

[185] Mancina RM Dongiovanni P Petta S Pingitore P Meroni M Rametta R. The MBOAT7-TMC4 variant rs641738 increases risk of nonalcoholic fatty liver disease in individuals of European descent. Gastroenterology. (2016) 150:1219–30.e6. 10.1053/j.gastro.2016.01.032 26850495 PMC4844071

[186] Luukkonen PK Zhou Y Hyötyläinen T Leivonen M Arola J Orho-Melander M. The MBOAT7 variant rs641738 alters hepatic phosphatidylinositols and increases severity of non-alcoholic fatty liver disease in humans. J Hepatol. (2016) 65:1263–5. 10.1016/j.jhep.2016.07.045 27520876

[187] Krawczyk M Rau M Schattenberg JM Bantel H Pathil A Demir M. Combined effects of the PNPLA3 rs738409, TM6SF2 rs58542926, and MBOAT7 rs641738 variants on NAFLD severity: a multicenter biopsy-based study. J Lipid Res. (2017) 58:247–55. 10.1194/jlr.P067454 27836992 PMC5234727

[188] Umano GR Caprio S Di Sessa A Chalasani N Dykas DJ Pierpont B. The rs626283 variant in the MBOAT7 gene is associated with insulin resistance and fatty liver in caucasian obese youth. Am J Gastroenterol. (2018) 113:376–83. 10.1038/ajg.2018.1 29485130 PMC12136689

[189] Ross MG Desai M. Developmental programming of offspring obesity, adipogenesis, and appetite. Clin Obstet Gynecol. (2013) 56:529–36. 10.1097/GRF.0b013e318299c39d 23751877 PMC4191824

[190] Brandt S Roos J Inzaghi E Kotnik P Kovac J Battelino T. Circulating levels of miR-122 and nonalcoholic fatty liver disease in pre-pubertal obese children. Pediatr Obes. (2018) 13:175–82. 10.1111/ijpo.12261 29271122

[191] Esau C Davis S Murray SF Yu XX Pandey SK Pear M. miR-122 regulation of lipid metabolism revealed by *in vivo* antisense targeting. Cell Metab. (2006) 3:87–98. 10.1016/j.cmet.2006.01.005 16459310

[192] Yamada H Ohashi K Suzuki K Munetsuna E Ando Y Yamazaki M. Longitudinal study of circulating miR-122 in a rat model of non-alcoholic fatty liver disease. Clin Chim Acta. (2015) 446:267–71. 10.1016/j.cca.2015.05.002 25958847

[193] Gerhard GS Malenica I Llaci L Chu X Petrick AT Still CD. Differentially methylated loci in NAFLD cirrhosis are associated with key signaling pathways. Clin Epigenetics. (2018) 10:93. 10.1186/s13148-018-0525-9 30005700 PMC6044005

[194] Murphy SK Yang H Moylan CA Pang H Dellinger A Abdelmalek MF. Relationship between methylome and transcriptome in patients with nonalcoholic fatty liver disease. Gastroenterology. (2013) 145:1076–87. 10.1053/j.gastro.2013.07.047 23916847 PMC3805742

[195] Huebert RC Vasdev MM Shergill U Das A Huang BQ Charlton MR. Aquaporin-1 facilitates angiogenic invasion in the pathological neovasculature that accompanies cirrhosis. Hepatology. (2010) 52:238–48. 10.1002/hep.23628 20578142 PMC2928054

[196] Jin-no K Tanimizu M Hyodo I Kurimoto F Yamashita T. Plasma level of basic fibroblast growth factor increases with progression of chronic liver disease. J Gastroenterol. (1997) 32:119–21. 9058307 10.1007/BF01213308

[197] Lynch C Chan CS Drake AJ. Early life programming and the risk of non-alcoholic fatty liver disease. J Dev Orig Health Dis. (2017) 8:263–72. 10.1017/S2040174416000805 28112071

[198] McCurdy CE Bishop JM Williams SM Grayson BE Smith MS Friedman JE. Maternal high-fat diet triggers lipotoxicity in the fetal livers of nonhuman primates. J Clin Invest. (2009) 119:323–35. 10.1172/JCI32661 19147984 PMC2631287

[199] Modi N Murgasova D Ruager-Martin R Thomas EL Hyde MJ Gale C. The influence of maternal body mass index on infant adiposity and hepatic lipid content. Pediatr Res. (2011) 70:287–91. 10.1203/PDR.0b013e318225f9b1 21629154

[200] Brumbaugh DE Tearse P Cree-Green M Fenton LZ Brown M Scherzinger A. Intrahepatic fat is increased in the neonatal offspring of obese women with gestational diabetes. J Pediatr. (2013) 162:930–6.e1. 10.1016/j.jpeds.2012.11.017 23260099 PMC3610780

[201] Ayonrinde OT Oddy WH Adams LA Mori TA Beilin LJ de Klerk N. Infant nutrition and maternal obesity influence the risk of non-alcoholic fatty liver disease in adolescents. J Hepatol. (2017) 67:568–76. 10.1016/j.jhep.2017.03.029 28619255

[202] Nguyen LT Chen H Zaky A Pollock C Saad S. SIRT1 overexpression attenuates offspring metabolic and liver disorders as a result of maternal high-fat feeding. J Physiol. (2019) 597:467–80. 10.1113/JP276957 30381838 PMC6332732

[203] Aagaard-Tillery KM Grove K Bishop J Ke X Fu Q McKnight R. Developmental origins of disease and determinants of chromatin structure: maternal diet modifies the primate fetal epigenome. J Mol Endocrinol. (2008) 41:91–102. 10.1677/JME-08-0025 18515302 PMC2959100

[204] Thorn SR Baquero KC Newsom SA El Kasmi KC Bergman BC Shulman GI. Early life exposure to maternal insulin resistance has persistent effects on hepatic NAFLD in juvenile nonhuman primates. Diabetes. (2014) 63:2702–13. 10.2337/db14-0276 24705404 PMC4113070

[205] Troisi J Pierri L Landolfi A Marciano F Bisogno A Belmonte F. Urinary metabolomics in pediatric obesity and NAFLD identifies metabolic pathways/metabolites related to dietary habits and gut-liver axis perturbations. Nutrients. (2017) 9:E485. 10.3390/nu9050485 28492501 PMC5452215

[206] Pandey KB Rizvi SI. Plant polyphenols as dietary antioxidants in human health and disease. Oxid Med Cell Longev. (2009) 2:270–8. 10.4161/oxim.2.5.9498 20716914 PMC2835915

[207] Amo K Arai H Uebanso T Fukaya M Koganei M Sasaki H. Effects of xylitol on metabolic parameters and visceral fat accumulation. J Clin Biochem Nutr. (2011) 49:1–7. 10.3164/jcbn.10-111 21765599 PMC3128359

[208] Elliott P Posma JM Chan Q Garcia-Perez I Wijeyesekera A Bictash M. Urinary metabolic signatures of human adiposity. Sci Transl Med. (2015) 7:285ra62. 10.1126/scitranslmed.aaa5680 25925681 PMC6598200

[209] Butte NF Liu Y Zakeri IF Mohney RP Mehta N Voruganti VS. Global metabolomic profiling targeting childhood obesity in the Hispanic population. Am J Clin Nutr. (2015) 102:256–67. 10.3945/ajcn.115.111872 26085512 PMC4515872

[210] Troisi J Belmonte F Bisogno A Lausi O Marciano F Cavallo P. Salivary markers of hepato-metabolic comorbidities in pediatric obesity. Dig Liver Dis. (2019) 51:516–23. 10.1016/j.dld.2018.11.009 30528710

[211] Pacifico L Cantisani V Anania C Bonaiuto E Martino F Pascone R. Serum uric acid and its association with metabolic syndrome and carotid atherosclerosis in obese children. Eur J Endocrinol. (2009) 160:45–52. 10.1530/EJE-08-0618 18952765

[212] Nejatinamini S Ataie-Jafari A Qorbani M Nikoohemat S Kelishadi R Asayesh H. Association between serum uric acid level and metabolic syndrome components. J Diabetes Metab Disord. (2015) 14:70. 10.1186/s40200-015-0200-z 26380228 PMC4570526

[213] Troisi J Belmonte F Bisogno A Pierri L Colucci A Scala G. Metabolomic salivary signature of pediatric obesity related liver disease and metabolic syndrome. Nutrients. (2019) 11:E274. 10.3390/nu11020274 30691143 PMC6412994

[214] Wasilewska N Bobrus-Chociej A Harasim-Symbor E Tarasów E Wojtkowska M5 Chabowski A. Increased serum concentration of ceramides in obese children with nonalcoholic fatty liver disease. Lipids Health Dis. (2018) 17:216. 10.1186/s12944-018-0855-9 30208901 PMC6136227

[215] Qi S Xu D Li Q Xie N Xia J Huo Q. Metabonomics screening of serum identifies pyroglutamate as a diagnostic biomarker for nonalcoholic steatohepatitis. Clin Chim Acta. (2017) 473:89–95. 10.1016/j.cca.2017.08.022 28842175

